# Non‐invasive brain stimulation of motor cortex induces embodiment when integrated with virtual reality feedback

**DOI:** 10.1111/ejn.13871

**Published:** 2018-03-09

**Authors:** M. Bassolino, M. Franza, J. Bello Ruiz, M. Pinardi, T. Schmidlin, M.A. Stephan, M. Solcà, A. Serino, O. Blanke

**Affiliations:** ^1^ Laboratory of Cognitive Neuroscience Brain Mind Institute Ecole Polytechnique Fédérale de Lausanne Geneva Switzerland; ^2^ Center for Neuroprosthetics School of Life Sciences Ecole Polytechnique Fédérale de Lausanne 9 Chemin des Mines 1202 Geneva Switzerland; ^3^ Center for Neuroprosthetics School of Life Sciences Ecole Polytechnique Fédérale de Lausanne Clinique Romande de Réadaptation Sion Switzerland; ^4^ MySpace Lab Department of Clinical Neurosciences University Hospital Lausanne (CHUV) Lausanne Switzerland; ^5^ Department of Neurology University of Geneva Geneva Switzerland

**Keywords:** hand corticospinal tract, ownership, rubber hand illusion, transcranial magnetic stimulation, virtual reality

## Abstract

Previous evidence highlighted the multisensory‐motor origin of embodiment – that is, the experience of having a body and of being in control of it – and the possibility of experimentally manipulating it. For instance, an illusory feeling of embodiment towards a fake hand can be triggered by providing synchronous visuo‐tactile stimulation to the hand of participants and to a fake hand or by asking participants to move their hand and observe a fake hand moving accordingly (rubber hand illusion). Here, we tested whether it is possible to manipulate embodiment not through stimulation of the participant's hand, but by directly tapping into the brain's hand representation via non‐invasive brain stimulation. To this aim, we combined transcranial magnetic stimulation (TMS), to activate the hand corticospinal representation, with virtual reality (VR), to provide matching (as contrasted to non‐matching) visual feedback, mimicking involuntary hand movements evoked by TMS. We show that the illusory embodiment occurred when TMS pulses were temporally matched with VR feedback, but not when TMS was administered outside primary motor cortex, (over the vertex) or when stimulating motor cortex at a lower intensity (that did not activate peripheral muscles). Behavioural (questionnaires) and neurophysiological (motor‐evoked‐potentials, TMS‐evoked‐movements) measures further indicated that embodiment was not explained by stimulation *per se*, but depended on the temporal coherence between TMS‐induced activation of hand corticospinal representation and the virtual bodily feedback. This reveals that non‐invasive brain stimulation may replace the application of external tactile hand cues and motor components related to volition, planning and anticipation.

## Introduction

The brain constantly receives, sends and updates information from and to the body, thus building association rules between different multisensory bodily signals (i.e. tactile, proprioceptive, kinesthetic, visual, auditory, vestibular), motor commands and related external events (e.g. Medina & Coslett, 2010; Serino & Haggard, 2010; De Vignemont, 2011; Held *et al*., 2011). Integrated signals between motor intention, execution and multisensory feedback have been proposed to lead to a sense of control for one's own movements (sense of agency, ‘I am the one who generated that hand movement’) and to a feeling of ownership for one's own body (‘the hand which is moving is my hand’). The sense of ownership and sense of agency are fundamental components of embodiment (i.e. the experience of having a body and being in control of it), and several experimental procedures to manipulate embodiment have been described (e.g. Jeannerod, 2003; Kannape & Blanke, 2013; Blanke *et al*., 2015). For instance, embodiment for a fake hand can be induced through multisensory stimulation using the rubber hand illusion (RHI) (Botvinick & Cohen, 1998; Tsakiris, 2010). During the RHI, synchronous visuo‐tactile stimulation is applied and participants observe touches on a fake hand, while receiving concurrent tactile stimuli on their hidden hand. Many other RHI‐like protocols have been proposed, such as by providing visuo‐motor stimulation based on participants' movements and congruent visual feedback of a fake or a virtual hand moving accordingly (e.g. Slater *et al*., 2008; Sanchez‐Vives *et al*., 2010; Kilteni *et al*., 2015). Thus, most RHI work has been based either on the direct application of somatosensory stimuli to participant's skin or limb (i.e. tactile, Botvinick & Cohen, 1998 or proprioceptive cue, Walsh *et al*., 2011) or on subjects' movements (Tsakiris *et al*., 2006; Riemer *et al*., 2013, 2014; Kalckert & Ehrsson, 2014).

One intriguing possibility is to induce embodiment by providing artificial stimulation able to activate the corticospinal hand representation. Despite recent clinical evidence in this direction (in two epileptic patients undergoing invasive stimulation of somatosensory cortex evoking artificial somatic sensations in patients' hand; Collins *et al*., 2017), the possibility to induce hand embodiment in healthy participants by artificially activating the corticospinal hand representation non‐invasively, that is through transcranial magnetic stimulation (TMS), has been never investigated, so far.

Here, we linked the stimulation of the hand corticospinal representation by non‐invasive transcranial magnetic stimulation (TMS) over the motor cortex (M1) with visual hand feedback provided by an immersive VR system (TMS‐VR induced RHI) to investigate embodiment induction for a virtual hand. If applied over hand M1, a TMS pulse, at a sufficient intensity, induces measurable twitches and short involuntary hand movements and in the corresponding hand muscle (i.e. motor‐evoked potentials, MEPs). In our new experimental set‐up, TMS automatically triggered the VR system, so that participants observed an animated virtual hand mimicking the TMS‐induced movements. We hypothesized that the combination between the activation of the hand corticospinal representation (induced through TMS), leading to involuntary hand twitches, time‐locked with visual feedback provided via VR (synchronous condition), induces illusory embodiment for the virtual limb. Thus, we compared the strength of subjective feelings of ownership for the virtual hand against a control condition where TMS and visual feedback were decoupled (asynchronous condition). To study the specificity of these effects for the location and intensity of TMS, we run two further conditions, where we applied TMS over a site outside M1 (vertex, supra‐threshold vertex) or over M1, but with reduced intensity (subthreshold condition, 80% of resting motor threshold), not evoking muscle contractions or peripheral movements.

## Materials and methods

### Subjects

Thirty‐two subjects were recruited (mean age 29.7 years, SD ± 4.7, 16 females). They all performed the main experimental condition (supra‐threshold M1 stimulation, 130% of the resting motor threshold) and in addition one of two other conditions. A first group of 16 subjects (mean age 29.8 years, SD ± 4.8, 8 females) underwent the ‘vertex’ control condition (supra‐threshold vertex), to test the alternative hypothesis that unspecific TMS effects unrelated to the activation of the M1 could induce embodiment for the virtual hand (experiment 1). The second group of 16 participants (mean age 29.6 years, SD ± 4.6, eight females) underwent the ‘subthreshold’ control condition (subthreshold M1), in which TMS over M1 was set not to evoke any muscle twitches or movements (experiment 2).

All participants were right‐handed, as determined by the Flinders Handedness survey (Nicholls *et al*., 2013). They had normal or corrected‐to‐normal vision, touch and hearing and no contraindication to TMS (Rossi *et al*., 2009). Participants were naive to the purpose of the study and participated after giving an informed consent. The study was conducted with the approval of the local ethics committee (Commission Cantonale Valaisanne d'Ethique Médicale, CCVEM 017/14).

### General procedure

**Figure 1:**
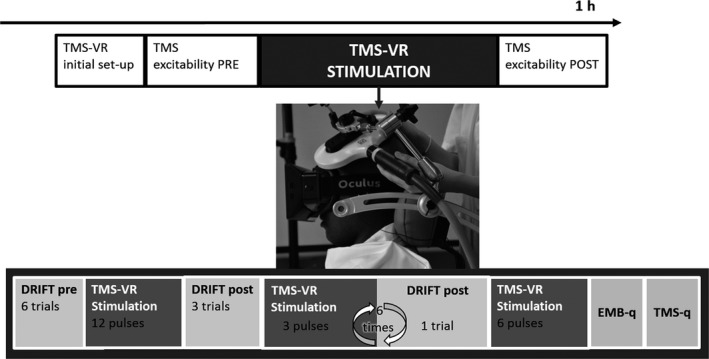
Transcranial Magnetic Stimulation (TMS) and virtual reality (VR) were fully integrated to induce rubber hand illusion (RHI): TMS‐VR induced RHI. It shows the experimental procedure: after the initial set‐up for TMS and VR (see main text), excitability of motor cortex was recorded at rest before and after the TMS‐VR stimulation (white squares). During the TMS‐VR stimulation (grey squares), participants received single TMS pulses, over motor cortex or the vertex, and at a specific intensity of stimulation, supra‐ or subthreshold, accordingly to the experimental conditions. Through a head‐mounted display (small photo), they observed a virtual hand mimicking the TMS‐evoked movements. The temporal congruency between TMS pulse and the movement of the virtual hand was manipulated in a synchronous or asynchronous condition. Before and after the first block of TMS‐VR stimulation (12 TMS pulses), participants performed the hand location task in VR (drift). Then, brief blocks of TMS‐VR stimulation (three pulses) followed by a drift measure were repeated six times. At the end, participants responded to a questionnaire related to embodiment (Embodiment questionnaire, in the figure EMB‐q) and to one related to the sensations induced by the TMS (TMS questionnaire, in the figure TMS‐q). This procedure was repeated twice in the same session, once for synchronous and once for asynchronous condition (in a counterbalanced between‐participants order). On a separate day, a second identical session was performed, which differed for the site (experiment 1) or the intensity (experiment 2) of TMS according to the experimental condition.

The experimental procedure is illustrated in Fig. 1. Subjects were seated in a comfortable TMS chair (Brainsight, Rogue Research) with their arms resting in a prone position on a white table. First, all TMS parameters were set: the area to stimulate, the intensity of the stimulation and the movements evoked (see below). Next, participants were familiarized with the VR scenario by wearing a head‐mounted display (see below for details about VR): they were instructed to keep their right hand still and as relaxed as possible and to observe the virtual hand lying palm down on a white table. In an initial calibration phase, performed for every subject and experimental session, the position of the virtual hand was carefully set in order to match the perceived position of the participants’ right limb.

After VR calibration, we first recorded motor‐evoked potentials (MEPs, 12), as a control measure for cortical excitability at baseline, while participants observed a neutral virtual scenario (a white table). After this, participants performed a hand location task (drift PRE, six trials; see below). At that time, they underwent the TMS‐VR stimulation for about 2 min (12 TMS pluses). Next, participants repeated the drift (POST, three trials). Then, short blocks of TMS‐VR stimulation (three TMS pulses, approximately 30 s) were followed by one trial of the drift. This loop was repeated six times. After this, another TMS‐VR stimulation followed (six TMS pulses, approximately 1 min) and at the end of this block of stimulation, participants were asked to answer to two questionnaires (see below), one related to embodiment (Embodiment questionnaire) and one regarding the sensations induced by TMS (TMS questionnaire).

Half of the subjects (16 subjects in total, balanced in supra‐threshold vertex and subthreshold M1 conditions) performed the Embodiment questionnaire after the drift‐stimulation loop as just described, while the other half (16) performed first the Embodiment questionnaire and then the loop. The TMS questionnaire was always performed at the end of the entire procedure. After the TMS questionnaire, we recorded MEPs (12 trials), as control measure for cortical excitability after the TMS‐VR stimulation, while participants observed the virtual white table.

This overall procedure remained identical for all conditions. The subjects performed the main experimental condition (supra‐threshold M1) on 1 day and one additional condition (either supra‐threshold vertex or subthreshold M1, depending on the group) on a different day, in a counterbalanced order, and with an interval of approximately 1 week between the two testing days. In each session, participants were exposed both to a condition in which TMS and VR feedback were time‐locked (synchronous condition) and to a condition in which a delay was inserted between the TMS pulses and visual feedback provided by the VR system (asynchronous condition, see below for details). There was a 10‐min break between the two stimulation blocks. The order between conditions and blocks was counterbalanced across participants.

### Transcranial magnetic stimulation

TMS was delivered through a figure‐eight coil (wing diameter of 70 mm) connected to a single Magstim monophasic stimulator (Magstim 200^2^; Magstim Co., Whitland, UK). To determine the optimal position for activation of the right first dorsal interosseous (FDI) muscle (i.e. the scalp position from which the largest MEPs were elicited), the coil was positioned at an initial estimate 5 cm lateral and 1 cm anterior to the vertex (Groppa *et al*., 2012). TMS pulses at slightly supra‐threshold intensity were then applied by moving the coil in 0.5 cm steps around this initial estimate. Resting motor threshold of FDI muscle was determined according to standard procedure using the software based ‘adaptive method’ (Awiszus, 2011) (TMS Motor Threshold Assessment Tool, http://www.clinicalresearcher.org/software.htm) (Groppa *et al*., 2012). During the procedure to define resting motor threshold, participants were asked to relax their muscles and wore the head‐mounted display to observe a neutral virtual scenario (a white empty table, i.e. without the virtual hand).

During supra‐threshold M1 and supra‐threshold vertex conditions, the intensity of the TMS pulse was set at 130% of the resting motor threshold to ensure stable MEPs and TMS‐evoked movements. During subthreshold M1 condition, the intensity was set at 80% of the resting motor threshold, according to previous studies showing that this intensity is not sufficient to induce MEPs (peak‐to‐peak amplitude higher than 0.05 mV) in the hand (e.g. Nakamura *et al*., 1997). The absence of any MEPs at 80% of resting motor threshold has been verified at the beginning of the experiment for every subject, and the amplitude of MEPs during the entire experiment has been recorded.

During supra‐threshold M1 and subthreshold M1 conditions, the coil was placed on the optimal position for activation of the right FDI, while for the supra‐threshold vertex condition, the coil was centred over the vertex (e.g. Sandrini *et al*., 2011), at the electrode position Cz as defined by the International 10–20 system (Jasper, 1958). The coil was placed tangentially to the scalp with the handle pointing 45° postero‐laterally away from the midline during supra‐threshold M1 and subthreshold M1, while the handle was pointing 0° posterior during supra‐threshold vertex (Duecker *et al*., 2013; Case *et al*., 2016).

In all conditions, the optimal position of the coil was marked on the scalp with a pen to ensure the correct coil placement throughout the experiment. During the entire experiment, the coil was fastened to an articulated mechanical arm. The intertrial interval between two consecutive TMS pulses was randomly varying from 9.8 to 12.2 s to ensure no change in cortical excitability (Chen *et al*., 1997) and to avoid expectancy.

#### Motor‐evoked potentials (MEPs)

To assess the excitability of the motor system, we measured the peak‐to‐peak MEP amplitudes elicited by TMS in the FDI muscle before, during and after the illusion. MEPs were recorded by means of a surface electromyographic system (EMG) through wireless electrodes positioned on the FDI in a tendon‐belly configuration. EMG signals were amplified and band‐pass filtered (1 Hz to 1 kHz) by a Noraxon DTS amplifier (Velamed; GmbH, Köln, Germany). The signals were sampled at 3000 Hz, digitized using a laboratory interface (Power1401; Cambridge Electronics Design CED) and stored on a personal computer for display and later offline data analysis using the Signal software. During the initial TMS calibration and MEP registration (see above), each recording epoch lasted 1500 ms, from 300 ms before to 1200 ms after the TMS pulse. During the TMS‐VR illusion, we recorded the EMG traces continuously and a trigger was given to the Noraxon system for every TMS pulse. The absence of voluntary contractions was continuously verified by visual monitoring of the EMG signal. Trials with EMG background activity (peak‐to‐peak amplitude > 0.05 mV in the 100 ms preceding the TMS pulse) were excluded from analysis.

#### TMS‐evoked movements

To assess the similarity between the movements evoked by the TMS and the observed virtual movements, we recorded the TMS‐evoked hand movements by means of a three‐dimensional accelerometer fixed over the middle finger knuckle (Noraxon (Velamed; GmbH). Accelerometric data for the separate axes were acquired in parallel with the EMG data, at the same sampling frequency. Accelerometric data were filtered (0.4–100 Hz) and analysed by a custom‐made software written in MATLAB, following methods already proposed in previous works (Classen *et al*., 1998; Finisguerra *et al*., 2015). The acceleration modulus (i.e. the square root of the sum of the squares of the axes) was first computed for a 200‐ms window starting from TMS delivery. According to previous studies, we calculated the acceleration onset as the time when 5% of the peak acceleration was detected. Trials were included in the analysis if peak acceleration appeared between 20 and 55 ms after the TMS pulse (Finisguerra *et al*., 2015) and its amplitude was equal to or higher than 0.09 m/s^2^ in one axis (Classen *et al*., 1998). Given that the scope of this control measure was to verify whether the movements induced by TMS were mimicking the virtual ones, we focused on Z‐component being sensitive to capture hand‐closing movements similar to those displayed in VR. We compared the percentage of the movements indicating hand‐closing in all experimental conditions.

### Virtual reality (VR)

Participants wore a head‐mounted display (Oculus Rift Development Kit 1, 640 × 800 resolution per eye, 110° Field of View (nominal), refresh rate 60 Hz; Oculus VR, Menlo Park, CA, USA). An in‐house software (ExpyVR, EPFL, http://lnco.epfl.ch/expyvr, framework for designing and running experiments in virtual reality) was used for stimulus presentation, to collect subjects’ answers and to generate triggers for TMS pulses. For this later purpose, the laptop running the VR software was connected to the laboratory interface (Power1401; Cambridge Electronics Design CED), by means of a laptop‐parallel‐adapter‐card to send triggers to the TMS stimulators.

On the head‐mounted display, participants observed virtual right hand movements mimicking their own hand movements evoked by supra‐threshold M1 TMS pulses. For this purpose, we controlled a realistic three‐dimensional virtual hand lying on a virtual white table in real time. The virtual hand's movements were animated to move with the same kind of movements that would be evoked by the supra‐threshold M1 TMS pulses, every time that a trigger was sent to the TMS stimulators. During the TMS‐VR stimulation, the virtual hand was displaced leftward with respect to the perceived position of the real hand as defined in an initial calibration phase. The inclination of the real table was adjusted to match the subjects’ perceived inclination of the table in VR. In the synchronous condition, the animated movement of the virtual hand started right after the trigger was sent to the TMS stimulators to temporally match the real hand evoked movement. An intrinsic delay of about 65 ms, due to the connection between the TMS and VR systems, was however present but did not have any effect on the perceived synchronicity. Indeed, visual animation occurred before the value of 150 ms that is generally necessary for detecting visuo‐motor conflicts (Blakemore *et al*., 1999; Tsakiris *et al*., 2006) and it was longer than the physiological latency between TMS pulse and peripheral motor effects in the hand [in healthy young subjects, the latency of muscle twitches in the FDI muscle is usually around the 20 ms (Groppa *et al*., 2012), while the onset of evoked hand movements is usually between 20 and 55 ms after the TMS pulse (Finisguerra *et al*., 2015). In the asynchronous condition, three different delays of either 600, 1200 or 1800 ms were inserted between TMS pulse and visual VR feedback (the three delays were applied in random order and balanced between blocks and conditions). Importantly, when debriefing participants at the end of the experiment, they confirmed that (i) in the synchronous condition, the virtual hand movements appeared at the same time as the TMS pulses and as the perceived real hand movements, while (ii) in the asynchronous condition, they appeared as delayed with respect to the TMS pulses.

### RHI induced by coupling of TMS with VR (TMS‐VR‐induced RHI)

The general procedure remained identical in all conditions (see above), which differed for the site (M1 or vertex) or the intensity (supra‐threshold or subthreshold) of stimulation and the temporal congruency between virtual and real hand movements (synchronous and asynchronous).

We assessed the TMS‐VR‐induced RHI by means of consolidated measures previously used in RHI studies (Botvinick & Cohen, 1998; Tsakiris *et al*., 2006): (i) standard questionnaires assessing the subjective experience of embodiment (Embodiment questionnaire); and (ii) a hand location task evaluating the proprioceptive drift. Moreover, MEPs and TMS‐evoked movements were recorded during the whole duration of the stimulation to measure how cortical excitability and peripheral TMS effects varied across conditions.

#### Embodiment questionnaire

The items of the Embodiment questionnaire were selected from those used previously to test different embodiment components (Longo *et al*., 2008). Our main component of interest was related to ownership for the virtual hand (‘it seemed like the virtual hand was part of my body’; ‘it seemed like the virtual hand was my hand’). Considering previously reported dissociations between ownership and other embodiment‐related components (e.g. Longo *et al*., 2008; Serino *et al*., 2013), we also included in the questionnaire the following additional components: disownership for the physical hand (‘it seemed like my hand had disappeared’; ‘it seemed like the experience on my hand was less vivid than normal’);location (‘it seemed like the virtual hand was in the location where my hand was’; ‘it seemed like my hand was in the location where the virtual hand was’) and agency (‘it seemed like I was in control of the virtual hand’; ‘it seemed like I could have moved the virtual hand if I had wanted’). The questionnaire included two statements for each component, plus two control questions (‘it seemed like I had more than two hands’; ‘it seemed like I had three hands’) (10 items in total). The two statements referring to the same component (Longo *et al*., 2008) were collapsed together for the analysis.

Subjects were asked to indicate agreement or disagreement with the statements of the questionnaire using the keyboard to move a cursor on a continuous vertical scale displayed through the head‐mounted display. The top extreme of the scale, indicated by a green dot, represented a complete agreement (score 6), while the bottom extreme of scale, indicated by a red dot, corresponded to a complete disagreement (score 0).

#### Proprioceptive drift

The hand location task (proprioceptive drift) was similar to that described elsewhere (Tsakiris, 2010 for a review). Before and after the TMS‐VR stimulation, participants were asked to indicate the perceived position of their real right hand in VR using a keyboard with their left hand to move a cursor on a white empty table. Given that during the TMS‐VR stimulation the virtual hand was displaced with respect to the perceived position of participants’ real hand, we verified whether after the stimulation, participants reported the perceived position of their hand as drifted towards the location of the virtual hand.

#### Control measures

In order to assess our subjects’ perception to the different TMS conditions (supra‐threshold M1, supra‐threshold vertex, subthreshold M1), participants responded also to a *TMS questionnaire* about general sensations induced by the pulse (‘Did you perceive any sensation on your head induced by TMS?’; ‘Did you hear the click sounds induced by TMS?’) and about specific perceptions regarding the hand and the evoked movements (‘Did you perceive any sensation on your hand induced by TMS?’; ‘Did you perceive your hand moving?’). The general sensations induced by TMS are expected to be similar in all the conditions, because of the contact between the coil and the scalp and because of the TMS sound clicks. In contrast, the sensations regarding the hand are specific to the supra‐threshold M1 condition. Participants used the same continuous scale used for the Embodiment questionnaire to rate the intensity of the induced sensations (from very high, green dot, to very low, red dot). One subject's answers at TMS questionnaire were not recorded due to technical problems in experiment 1.

Moreover, *MEPs* and *TMS‐evoked movements* were recorded for the entire duration of the stimulation, as control measures of excitability and of the peripheral effects, respectively. One subject's accelerometric data were not recorded in experiment 1 due to a technical problem.

To reduce the perception of the click sound produced by TMS, during stimulation and also MEP recording, and to exclude surrounding auditory cues, participants were listening to white noise presented through noise‐cancelling ear plugs (Bose Quiet Comfort 20) during the experiment. For every subject, the sound volume was adjusted at the beginning of the session. Due to the different click sounds volume produced by TMS pulses at the different intensities of stimulation (i.e. lower sound volume in the subthreshold M1 versus higher sound volume in supra‐threshold M1), during the subthreshold M1 condition a second sham coil was connected to the stimulator in order to discharge at the same time of the real coil and mask any possible difference between the conditions. In addition, subjects’ perception of the TMS sound was assessed with the already described *TMS questionnaire*. Finally, to assure subjects’ attention during the TMS‐VR stimulation, they were requested to count red dots randomly appearing on the virtual index finger in the interval between two consecutive TMS pulses. The performance at this task was always very high (> 90% of correct responses).

#### Similarity between virtual stimuli and TMS‐evoked movements

To tests the protocol duration and to prepare the stimuli in VR, we performed preliminary tests (six subjects, about 140 TMS stimuli per subject in total considering the whole procedure). We recorded the movements evoked by TMS pulses applied at the same intensity and coil location as in the main experiment (supra‐threshold M1, 130% resting motor threshold, stimulation over the optimal position for the right FDI muscle activation, see above). Preliminary tests revealed that the movements typically evoked with that type of stimulation are a closing hand twitch, involving the whole hand, while in few cases (few trials in two subjects) an index abduction or extension occurred. Before the actual experiment, we inspected for every subject the TMS‐evoked movements, to select the most appropriate stimulus in VR. TMS induced a closing hand movement in almost all subjects of our sample (i.e. in 31 of 32 subjects).

### Statistical analysis

A repeated‐measures anovas were run on subjective ratings of the four components related to the embodiment (ownership, disownership, location, agency) with ‘embodiment components’ (four levels), ‘temporal congruency’ (synchronous vs. asynchronous) and ‘site’ (experiment 1, M1 vs. vertex) or ‘intensity’ (experiment 2, supra‐threshold vs. subthreshold) of stimulation as within‐subjects factors. Similar repeated‐measures anovas with the within‐subjects factors ‘temporal congruency’ and ‘site’ (experiment 1) or ‘intensity’ (experiment 2) of stimulation were performed on (i) the ratings related to the control component of the Embodiment questionnaire; (ii) on the perceived position of participants’ hand reported in the proprioceptive drift task (difference between post and pre); (iii) on ratings to the four items of the TMS questionnaire with the additional within‐subjects factor ‘sensations’ (four levels: sensations related to the hand, to the head, to the TMS‐evoked movements and to the sound clicks); (iv) on the MEPs amplitude before and after the stimulation with the supplementary within‐subject factor of ‘time’ (two levels: pre vs. post). If the anova revealed a significant interaction, we corrected for multiple comparisons using Newman–Keuls *post hoc* tests. The percentage of TMS‐evoked movements and the MEP amplitude during the stimulation were compared in synchronous versus asynchronous condition by means of paired *t*‐tests, Bonferroni corrected. An additional analysis about the supra‐threshold M1 stimulation putting together data from experiment 1 and from experiment 2 has been included in Appendix S1 (see also Fig. S1 and S2).

## Results

### Experiment 1: combining VR with supra‐threshold TMS over motor cortex, but not over the vertex, induces embodiment for a virtual hand

#### Embodiment questionnaire

**Figure 2:**
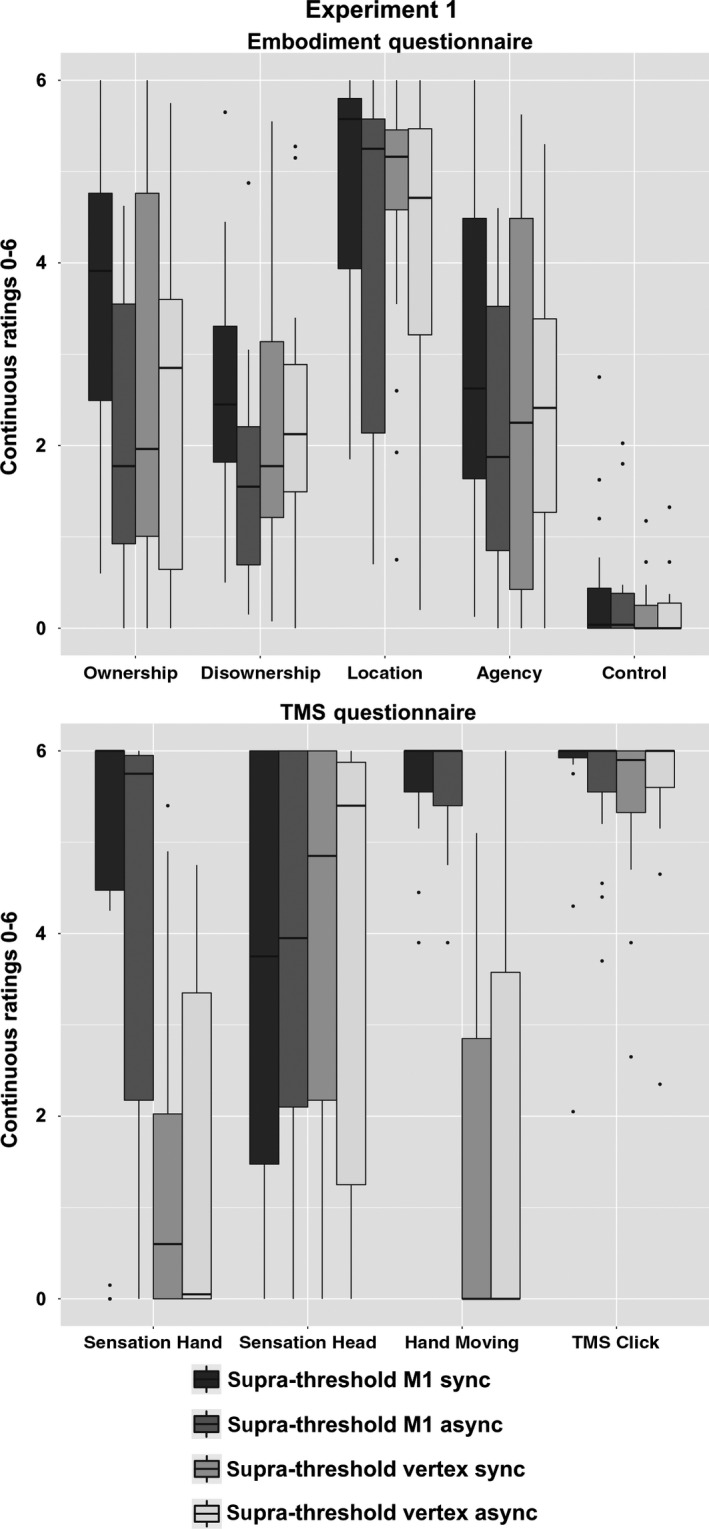
Experiment 1. Supra‐threshold transcranial magnetic stimulation (TMS) over the M1 vs. supra‐threshold TMS over the vertex. Figure shows results on subjective ratings at the Embodiment questionnaire (upper panel) in the supra‐threshold M1 vs. supra‐threshold vertex condition (16 subjects). ‘Boxes’ are based on the first and third quartiles (interquartile range, lower and upper ‘hinges’), the median (line), the largest and the smallest value no further than 1.5 × the interquartile range (upper and lower whiskers), data beyond the end of the whiskers (points). While higher ratings to the ‘embodiment questions’ after synchronous (in the figure, sync) rather than asynchronous (async) condition were reported in supra‐threshold M1, no synchronous–asynchronous difference was found in supra‐threshold vertex. This suggests that no illusion was induced after vertex stimulation. No synchronous–asynchronous or M1‐vertex difference emerged in the control questions. As expected, subjects’ ratings on sensations induced by TMS (TMS questionnaire, lower panel) on the hand (somatosensory sensation on the hand and perception of TMS‐induced movements) were different between supra‐threshold M1 and supra‐threshold vertex conditions, while general TMS sensations related to the somatosensory sensation on the head or the TMS sound clicks were not different between conditions.

We found a significant interaction ‘site of stimulation × temporal congruency’ (*F*_1,15_ = 5.82, *P* = 0.029), independently of the four components related to the Embodiment (‘site of stimulation × temporal congruency × embodiment components’: *F*_3,45_ = 0.99, *P* = 0.40). *Post hoc* test revealed higher ratings in the synchronous as compared to the asynchronous condition after M1 stimulation (*P* = 0.005) and not after vertex stimulation (*P* = 0.49). As expected, no synchronous–asynchronous difference emerged in the control questions, neither in M1 nor in vertex condition (main effect ‘temporal congruency’: *F*_1,15_ = 0.11, *P* = 0.75; ‘site of stimulation × temporal congruency’: *F*_1,15_ = 0.08, *P* = 0.78). These data show that supra‐threshold stimulation over M1 induces the illusory embodiment for the virtual hand selectively when combined with a temporally congruent visual feedback in virtual reality (see Fig. 2; means and standard errors are reported in Table S1, Supporting Information).

#### Drift

A positive drift towards the virtual hand was present across all conditions (always different from zero, *P* < 0.0125, alpha set at 0.05/4 = 0.0125 following Bonferroni correction), both in synchronous and asynchronous conditions and this occurred in the supra‐threshold M1 and supra‐threshold vertex conditions (main effect ‘temporal congruency’: *F*_1,15_ = 0.72, *P* = 0.41; interaction ‘site of stimulation × temporal congruency’: *F*_1,15_ = 0.52, *P* = 0.48).

#### TMS questionnaire

A significant interaction ‘site of stimulation × sensations’ was found (*F*_3,42_ = 19.76, *P* < 0.0001). Importantly, no differences between synchronous and asynchronous condition emerged (‘site of stimulation × temporal congruency × sensations’: *F*_3,42_ =0.69, *P* = 0.57; ‘site of stimulation × temporal congruency’: *F*_1,14_ = 0.08, *P* = 0.77). *Post hoc* test showed no difference in general TMS effects, such as the TMS‐induced somatosensory sensation on the head (*P* = 0.45) and the TMS sound clicks (*P* = 0.80), reported by participants during M1 and vertex stimulation. In contrast, as expected, higher ratings to hand sensations induced by TMS (*P* < 0.001) and the perception of the TMS‐evoked hand movements (*P* < 0.001) were found in M1 than in vertex stimulation (see Fig. 2; means and standard errors are reported in Table S1, Supporting Information). Altogether these data suggest that participants gave different ratings after M1 and vertex stimulation related to the hand effects evoked by the TMS (sensation and movement), but similar scores to the general TMS effects, such as the TMS‐induced somatosensory sensation on the head and the TMS sound clicks. Importantly, no difference was noticed between synchronous and asynchronous stimulation, thus indicating that the subjective changes found on embodiment in synchronous supra‐threshold M1 stimulation are not due to any intrinsic difference in sensation induced by synchronous and asynchronous conditions.

#### MEPs and TMS‐evoked movements

As expected, no movements were evoked during TMS over the vertex, while the percentage of evoked movements mimicking the hand movements observed in virtual reality during the supra‐threshold M1 was always very high in both the synchronous (mean = 92%, SE = 3.51) and asynchronous (mean = 90%, SE = 5.7) conditions [paired *t*‐test, *t*(14) = 0.75, *P* = 0.47]. Similarly, MEP amplitudes during synchronous (mean = 2.59 mV, SE = 0.45) and asynchronous (mean = 2.85 mV, SE = 0.52) condition were comparable in supra‐threshold M1 [paired *t*‐test, *t*(15) = −0.97, *P* = 0.35], while as expected no MEPs were recorded during the supra‐threshold vertex condition. Moreover, MEP amplitudes did not differ before and after supra‐threshold M1 and supra‐threshold vertex in both synchronous and a synchronous conditions (‘site of stimulation × temporal congruency × time’: *F*_1,15_ =0.061, *P* = 0.81, all main effects *P* > 0.05) (Fig. S3).

### Experiment 2: subthreshold TMS over M1 does not induce RHI

#### Embodiment questionnaire

**Figure 3:**
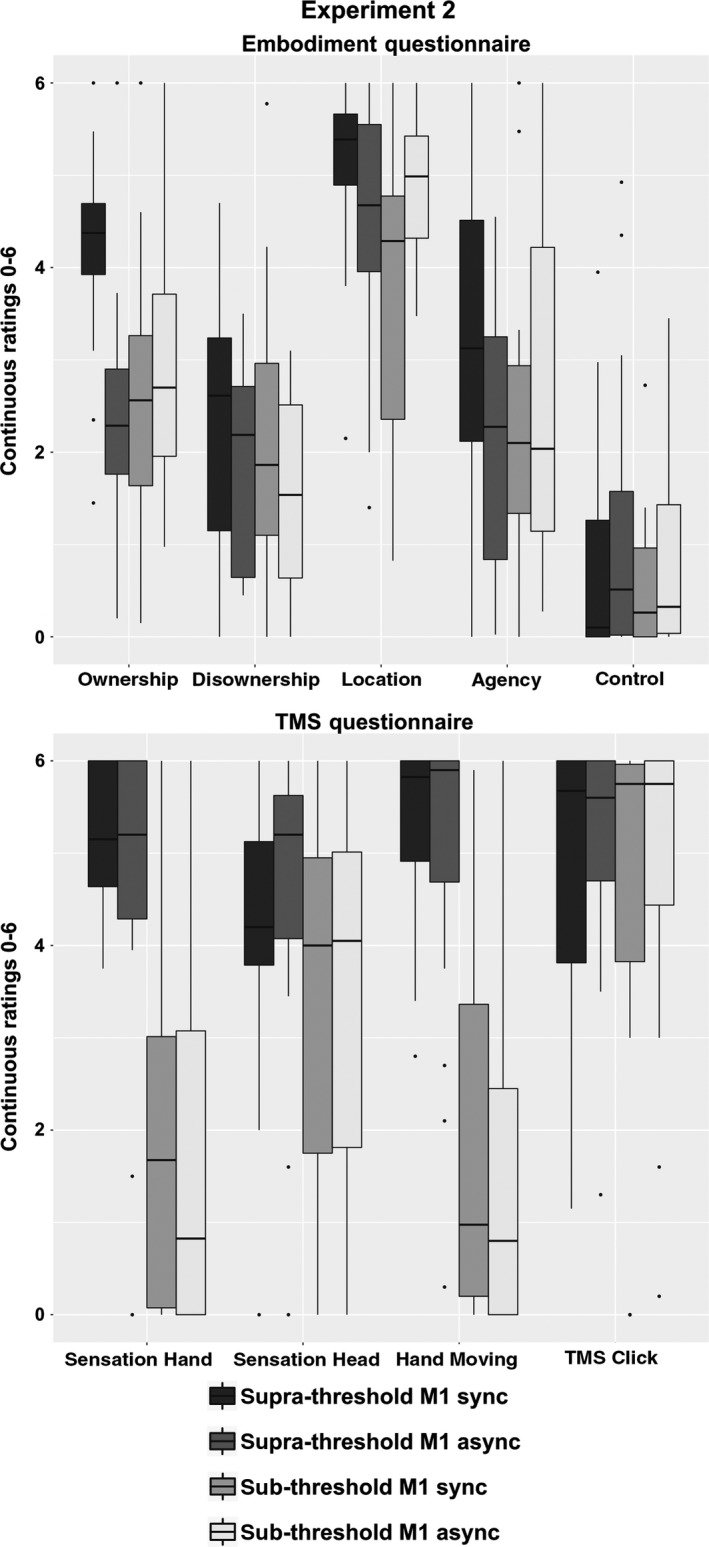
Experiment 2. Supra‐threshold vs. subthreshold transcranial magnetic stimulation (TMS) over M1. Figure shows findings related to subjective ratings (Embodiment questionnaire, upper panel) in the supra‐threshold M1 vs. subthreshold M1 condition (16 subjects). ‘Boxes’ are based on the first and third quartiles (interquartile range, lower and upper ‘hinges’), the median (line), the largest and the smallest value no further than 1.5 × the interquartile range (upper and lower whiskers), data beyond the end of the whiskers (points). While higher ratings after synchronous (in the figure, sync) rather than asynchronous (async) condition were reported in supra‐threshold M1 for the ‘ownership for the virtual hand’ and ‘agency’ component, this was not the case in subthreshold M1 condition. No synchronous–asynchronous or suprathreshold/subthreshold stimulation difference emerged in the control questions. As expected, and similar to experiment 1, subjects’ ratings on sensation induced by TMS (TMS questionnaire, lower panel) on the hand (somatosensory sensation on the hand and perception of TMS‐induced movements) were different between supra‐threshold M1 and subthreshold M1 conditions, while general TMS sensations related to somatosensory sensation on the head or the TMS sound clicks were not different between conditions.

We found a significant interaction ‘intensity of stimulation × temporal congruency × embodiment components’ (*F*_3,45_ =3.23, *P* = 0.031) on the ratings related to the embodiment. Thus, to understand the source of this interaction, we run four separate anovas on each embodiment component with ‘intensity of stimulation’ and ‘temporal congruency’ as within‐subjects factors. Significant 2 × 2 interactions were found for the main component of ownership (*F*_1,15_ = 12.19, *P* = 0.003), as well as for agency (*F*_1,15_ = 7.19, *P* = 0.017) and location statements (*F*_1,15_ = 9.73, *P* = 0.007). For both ownership and agency components, we found significantly higher ratings in the synchronous rather than in the asynchronous condition in the supra‐threshold, but not in the subthreshold stimulation (paired *t‐*test, ownership: supra‐threshold, *P* = 0.0004, subthreshold, *P* = 0.37; agency: supra‐threshold, *P* = 0.023, subthreshold, *P* = 0.28). Higher scores for location were found in the asynchronous rather than in the synchronous condition after the subthreshold stimulation, but not after the supra‐threshold one (supra‐threshold, *P* = 0.07; subthreshold, *P* = 0.01). Only a main effect of temporal congruency with no interaction statistically emerged on disownership, with higher score in the synchronous condition, suggesting that this effect was independent from the pattern of stimulation (main effect ‘temporal congruency’: *F*_1,15_ = 8.27, *P* = 0.012; ‘intensity of stimulation × temporal congruency’: *F*_1,15_ = 0.15, *P* = 0.70). As expected, no difference was found for the control questions in supra‐threshold or subthreshold conditions (main effect ‘temporal congruency’: *F*_1,15_ = 2.13, *P* = 0.17; ‘intensity of stimulation × temporal congruency’: *F*_1,15_ = 0.08, *P* = 0.79; see Fig. 3; means and standard errors are reported in Table S2, Supporting Information).

These data suggest that supra‐threshold, but not subthreshold M1 stimulation, induced illusory embodiment for the virtual hand when combined with synchronous visual feedback in virtual reality.

#### TMS questionnaire

A significant interaction ‘intensity of stimulation × sensations’ was found (*F*_3,45_ = 9.59, *P* < 0.0001). Importantly, no differences between synchronous and asynchronous condition emerged (‘intensity of stimulation × temporal congruency × sensations’: *F*_3,45_ = 0.37, *P* = 0.78; ‘intensity of stimulation × temporal congruency’: *F*_1,15_ = 0.13, *P* = 0.72). *Post hoc* test revealed that participants reported higher ratings in supra‐threshold M1 rather than subthreshold M1 at the TMS questionnaire about TMS‐induced hand sensations (*P* = 0.0001) and on the perception of the TMS‐induced movements (*P* = 0.0001). In contrast, similar ratings concerning the general effect of TMS were given during supra‐threshold M1 and subthreshold M1 (head sensation: *P* = 0.06; sound clicks: *P* = 0.81). This confirms that subjects rated differently the sensations related to the hand during supra‐threshold M1 and subthreshold M1 stimulation, despite similar scores reported to general effect of TMS (sensation on the head and sound clicks). Again, crucially, no synchronous–asynchronous difference emerged, thus excluding that the embodiment effects in the synchronous supra‐threshold M1 condition were due to intrinsic difference related to the stimulation in synchronous and asynchronous conditions (see Fig. 3; mean and standard error are reported in Table S2).

#### Drift

Regardless of the conditions (synchronous, asynchronous) and the intensity of stimulation (supra‐threshold M1; subthreshold M1), a positive drift towards the virtual hand was present (different from zero, Bonferroni corrected, *P* < 0.0125, alpha set at 0.05/4 = 0.0125) (main effect ‘temporal congruency’: *F*_1,15_ = 2.21, *P* = 0.16; interaction ‘intensity of stimulation × temporal congruency’: *F*_1,15_ = 0.008, *P* = 0.93).

#### MEPs and TMS‐evoked movements

As expected, movements or MEPs were evoked very rarely during subthreshold M1 stimulation (one movement of 36 delivered TMS pulses in two subjects, in synchronous and in asynchronous condition, respectively). In contrast during supra‐threshold M1, the percentage of evoked movements mimicking hand movements observed in virtual reality were very high in the synchronous (mean = 99%, SE = 0.36) and asynchronous (mean = 99%, SE = 0.31) conditions [percentage of movements: paired *t*‐test, *t*(15) = −0.15, *P* = 0.89]. The amplitude of MEPs was comparable in the synchronous (mean = 3.18 mV, SE = 0.76) and in the asynchronous (mean = 2.69 mV, SE = 0.50) conditions [paired *t*‐test, *t*(15) = 1.07, *P* = 0.30]. Moreover, MEP amplitude was equal before and after the supra‐threshold M1 or subthreshold M1 stimulation in both synchronous and asynchronous conditions (‘intensity of stimulation × temporal congruency × pre‐post’: *F*_1,15_ = 4.12, *P* = 0.06, all main effects *P* *>* 0.05) (Fig. S3).

## Discussion

The present findings demonstrate that a TMS supra‐threshold artificial signal over M1 triggering hand twitches, when combined with time‐locked visual feedback, is able to induce embodiment for a virtual hand in healthy participants. This novel form of RHI was driven by the temporal congruency between the TMS pulse activating the hand corticospinal tract and VR visual feedback and did not occur if TMS was administered outside M1 (over the vertex) or using an intensity of M1 stimulation below the motor threshold (subthreshold). This is the first report of the experimental induction of illusory embodiment using non‐invasive brain stimulation.

### Coupling TMS with VR to induce embodiment

While both VR and TMS have been previously applied in RHI protocols, this is the first study in which TMS is fully integrated with VR to induce embodiment. Several VR techniques have been employed to animate virtual hands based on tracked participants’ active movements (Sanchez‐Vives *et al*., 2010; Yuan & Steed, 2010; Kilteni *et al*., 2012). Previous studies used TMS to record the MEP amplitude as marker of ownership (Della Gatta *et al*., 2016) and of agency (Weiss *et al*., 2014) or to interfere with circumscribed cortical areas to investigate their role in RHI‐related processes (e.g. right temporo‐parietal junction, (Tsakiris *et al*., 2008); inferior posterior parietal lobule, (Kammers *et al*., 2009); left extrastriate body area, (Wold *et al*., 2014). Here, we directly coupled visual hand feedback via a fully controlled VR system with the TMS‐induced activation of the hand corticospinal tract to induce the RHI. The present effects confirm the possibility of inducing embodiment for a fake hand via brain stimulation and are in line with a recent proof‐of‐concept clinical study whereby the RHI was induced using synchronized cues between invasive S1 stimulation (via implanted subdural electrodes) and visual feedback provided manually to a physical fake hand in two epilepsy patients undergoing pre‐surgical epilepsy monitoring (Collins *et al*., 2017). Our approach further extends those findings by proposing a novel, non‐invasive, protocol that is applicable in healthy subjects and different patient populations (i.e. amputation, chronic pain, stroke), limits experimenter and subject biases (allowing double‐blind protocols), and enables fine‐grained control of a large number of experimental factors (various stimulation intensities and sites, different spatiotemporal couplings with VR feedback, and more complex VR scenarios).

### Inducing illusory embodiment by triggering artificial hand twitches through TMS

Previous work induced illusory feelings of embodiment for a virtual or fake hand by linking vision of the artificial limb with congruent bodily inputs of a real arm, using tactile and proprioceptive or motor information (Tsakiris, 2010; Blanke *et al*., 2015; Kilteni *et al*., 2015) or even sensory expectation (Ferri *et al*., 2013); here, we induced embodiment by artificially activating the hand corticospinal representation with TMS, without providing any direct cues on the physical hand. Moreover, the present protocol has several differences compared to earlier visuo‐motor version of RHI involving active and passive movements (Kalckert & Ehrsson, 2012, 2014; Riemer *et al*., 2013, 2014; Kilteni *et al*., 2015). First, the brief and involuntary TMS‐induced movements are weaker than prolonged, repetitive and large‐amplitude active movements and are smaller in amplitude and typically shorter than a passive movement (due to short‐lasting activity in corticospinal pathways of 10/15 ms (Gentner & Classen, 2006). Second, active movements are generally accompanied by voluntary motor commands, by motor planning and by anticipation (e.g. Wolpert & Ghahramani, 2000). As these latter aspects were absent (or at least strongly diminished), the present TMS‐RHI data show that these features characterizing active physical movements are not necessary to induce hand embodiment. Nevertheless, it is important to acknowledge that the afferent information evoked by TMS‐induced movements could be very similar to those evoked during passive movements. These inputs could have a role in triggering the illusion when combined with congruent visual feedback of a moving virtual hand (see below the comparison between supra‐ and sub threshold condition).

### Main features of the TMS‐VR induced RHI

The present supra‐threshold M1 stimulation when combined with temporally congruent virtual feedback induced an illusory feeling of ownership towards the virtual hand (experiment 1 and 2). Illusory embodiment involved all main components of hand embodiment as described for the RHI induced by manual stimulation protocols (Longo *et al*., 2008) (ownership, agency, location, disownership for the physical hand) when a larger sample size is considered (see the analysis in 32 subjects, Appendix S1 and Fig. S1).

The lack of any effects on control questions excludes compliance or suggestibility confounds. Crucially, the RHI occurred only when supra‐threshold TMS pulses over M1 were temporally linked with a virtual hand movement (synchronous), but not when a temporal delay was inserted between the TMS pulse and the movement of the hand shown on the head‐mounted display (asynchronous). This confirms previous RHI findings about the critical role of temporal congruency between the visual fake hand stimulus and the movement of the participant's hand (Tsakiris *et al*., 2006; Riemer *et al*., 2013, 2014) or tactile (Tsakiris, 2010) or cardiac (Suzuki *et al*., 2013) cues. Our data exclude that the RHI is caused by any difference in the perception of the TMS across these conditions, as indicated by the TMS questionnaire. Importantly, we also found no differences in TMS‐evoked movements or MEPs amplitude between synchronous and asynchronous supra‐threshold M1 conditions (see Fig. S3). Thus, subjective perceptions (TMS questionnaire), behavioural (TMS‐evoked movements) and neurophysiological (MEPs) data comprehensively argue against a role of the movement *per se* in explaining the present embodiment effects, but confirm the role of temporal congruency.

Moreover, our data show that the experimental induction of illusory embodiment is anatomically specific, in that it occurred only when TMS pulses were administered over M1, only in the synchronous condition, and not when TMS was applied over the vertex, ruling out the possibility that any generic effect of TMS (such as the sound clicks or any sensation on the scalp induced by the stimulation) and the temporal congruency between TMS and visual feedback could induce the illusion. In addition, no illusory embodiment was elicited by applying a subthreshold TMS pulse over M1 that did not evoke any sensory or movement effects at the hand (no MEPs, no muscle twitches), further underlining the selectivity of the obtained embodiment effects for the activation of the hand corticospinal representation. This shows that the illusory ownership in the present study was only triggered if hand twitches are evoked by the TMS and are combined with congruent visual feedback of a virtual hand moving accordingly to the evoked movements (synchronous condition). Alternatively, one could hypothesize that the subthreshold TMS intensity was not sufficient to activate M1 in order to trigger the illusion (in line with studies showing differences in M1 activation depending on TMS intensity, e.g. Shitara *et al*., 2011). At present, we do not exclude the possibility that slightly higher subthreshold stimulation (e.g. around the resting motor threshold) could induce illusory ownership even without inducing muscle twitches, an issue that should be investigated in future studies.

Finally, the same proprioceptive drift was present in all conditions as compared to baseline (compatible with condition‐independent high ratings of the ‘location’ item; Embodiment questionnaire), although the virtual hand was presented as displaced with respect to the perceived position of participants’ real hand as in earlier RHI studies (e.g. Tsakiris, 2010). Previous findings on the drift after RHI based on active and passive movements, however, reported condition‐specific drift (Tsakiris *et al*., 2006; Kammers *et al*., 2010; Sanchez‐Vives *et al*., 2010; Riemer *et al*., 2013; Kalckert & Ehrsson, 2014). We argue that this lack of synchronous–asynchronous difference of the drift might be related to strong visual capture of proprioception due to the high level of immersion of the VR system, and in line with other studies, it was independent of the pattern of stimulation (Kilteni *et al*., 2015) and the ownership modulation (Longo *et al*., 2008; Serino *et al*., 2013).

## Conclusion

The state of the motor system and a limb's current state of embodiment are mutually tied (Miller & Farné, 2016). On the one side, sensorimotor information together with available multisensory inputs is crucial to enable the sense of body ownership and agency (De Vignemont, 2011; Blanke *et al*., 2015; Bolognini *et al*., 2015; Kilteni *et al*., 2015). On the other side, embodiment modifications have been shown to affect motor excitability of the hand at rest (della Gatta *et al*., 2016) or during action observation (Schütz‐Bosbach *et al*., 2006, 2009). Here, we further extend this concept by showing that is possible to induce embodiment for a virtual hand by activating the corticospinal sensorimotor system via TMS, without motor‐related components of volition, planning or anticipation. The present form of non‐invasive brain stimulation over hand M1 coupled to VR can replace the application of tactile cues on the hand's skin (as typically used in the RHI), minimizes associated movements (only involuntary, small‐amplitude artificial movements), and fully automatizes the coupling between brain stimulation and visual feedback (mediated by the TMS‐coupled VR system). We argue that illusory embodiment in the present experiments is due to neuro‐visual integration between TMS‐induced M1 activation (and connections with sensory cortex, e.g. Jacobs *et al*., 2012) and hand twitches with visual VR feedback. Finally, our protocol may be clinically relevant for the evaluation and treatment of motor and embodiment disorders (Berti *et al*., 2005; Schwoebel & Coslett, 2005; Vallar & Ronchi, 2009; Bartolo *et al*., 2014; Bassolino *et al*., 2015; Bolognini *et al*., 2015), avoiding direct application of bodily cues and movement that may be perceived as painful and limit therapeutic options (i.e. allodynia in complex regional pain syndrome).

## Conflict of interest

The authors report no commercial or competing interests.

## Data accessibility

The main relevant data can be freely accessed at the following link: https://www.dropbox.com/sh/4vkzfgdnu68m6e2/AACsWUZBZ2ZOvmqJLcBLJ6tTa?dl=0

## Authors contribution

MB, MAS, AS and OB designed the study; MB, MF, MAS, MP and TS ran the experiments; MB, MF and MS analysed the data; JBR implemented the virtual reality‐brain stimulation set‐up. MB, AS and OB wrote the manuscript with assistance from other authors.

## Abbreviations

FDI: first dorsal interosseous muscle
M1: primary motor cortex
MEPs: motor‐evoked potentials
RHI: rubber hand illusion
TMS: transcranial magnetic stimulation
VR: virtual reality

## Supporting information


** **

Appendix S1. Additional analysis: supra‐threshold M1 in experiment 1 and 2.
Fig. S1. Main experiment.
Fig S2. Motor evoked potentials (MEPs) did not differ in synchronous or asynchronous condition.
Fig. S3. Motor evoked potentials (MEPs) in experiment 1 and 2.
Table S1. Experiment 1 (supra‐threshold M1 vs. supra threshold vertex).
Table S2. Experiment 2 (supra‐threshold M1 vs. sub‐threshold M1).
