# Supplementary material for: Non‐invasive brain stimulation of motor cortex induces embodiment when integrated with virtual reality feedback
Source: Eur J Neurosci. 2018 Mar 9;47(7):790–9. doi: 10.1111/ejn.13871 (PMC5900900; doi:10.1111/ejn.13871)
Supplement: Supplementary file 2 — Appendix S1. Additional analysis: supra‐threshold M1 in experiment 1 and 2. Fig. S1. Main experiment. Fig S2. Motor evoked potentials (MEPs) did not differ in synchronous or asynchronous condition. Fig. S3. Motor evoked potentials (MEPs) in experiment 1 and 2. Table S1. Experiment 1 (supra‐threshold M1 vs. supra threshold vertex). Table S2. Experiment 2 (supra‐threshold M1 vs. sub‐threshold M1). [file EJN-47-790-s002.pdf]

Supplementary Information

Additional analysis: supra-threshold M1 in experiment 1 and 2

We analyzed the data of the 32 subjects who were exposed to the TMS-VR induced RHI by applying supra-threshold TMS stimulation (130% of resting motor threshold) over M1 in experiment 1 (16 subjects) and 2 (16 subjects).

We compared the effects induced by supra-threshold stimulation when the movement of the virtual hand was temporally congruent (synchronous) or delayed (asynchronous) with respect to the TMS pulse. We applied the same statistical analysis used in experiment 1 and 2, with the exception of the factor "intensity" or "site" of stimulation that was absent here.

Embodiment-questionnaire. We found a significant interaction "temporal congruency X embodiment components" ($F(3, 93)=3.64$, $p=0.016$) on the ratings related to the embodiment. Post hoc tests revealed significantly higher ratings in the synchronous versus asynchronous conditions for all embodiment components (ownership: $p=0.0001$, disownership: $p=0.0496$, location: $p=0.008$, agency: $p=0.001$). The interaction was driven by the fact that the ratings among the 4 components were significantly different in the synchronous conditions (ownership > disownership: $p=0.0001$; ownership > agency: $p=0.014$; but ownership < location: $p=0.003$) while in the asynchronous condition the ratings were similarly low in the ownership, disownership and agency components (ownership versus disownership: $p=0.11$; ownership versus agency: $p=0.45$), with the exception of higher score in the location rather than in ownership (ownership < location: $p=0.001$).

As expected, responses to the control questions did not differ across conditions (paired t-test, $t(31)=-0.68$, $p=0.50$). In line with results of experiment 1 and 2, this analysis suggests

that supra-threshold M1 stimulation activating the hand corticospinal representation induces embodiment for the virtual hand if combined with synchronous visual feedback in virtual reality (Supplementary Figure 1).

TMS-q. No differences were found between synchronous and asynchronous conditions in subjects' ratings to sensations induced by TMS assessed through the TMS-questionnaire ("temporal congruency": $F(1, 30)=0.20$, $p=0.66$; "temporal congruency X questions": $F(3, 90)=2.34$, $p=0.08$). This suggests that the different effects found on embodiment in the synchronous and asynchronous conditions are not due to any intrinsic difference in the stimulation conditions (Supplementary Figure 1).

Drift. A positive drift towards the virtual hand position versus baseline was present in all conditions (synchronous: mean=0.68, SE=0.11, different from zero, $p>0.0001$; asynchronous: mean=0.62, SE=0.12, different from zero, $p>0.0001$), without any significant difference between synchronous and asynchronous (paired t-tests, $t(31)=0.41$, $p=0.69$).

MEPs and TMS-evoked movements. During stimulation, the percentage of TMS-evoked movements was high and, importantly, not different between synchronous (mean=96%, SE= 1.76) and asynchronous (mean=95%, SE=2.54) conditions (paired t-test, $t(30)=0.72$, $p=0.47$). Similarly, during stimulation, the MEP amplitudes did not differ either (synchronous: mean=2.88 mV, SE=0.44; asynchronous: mean=2.77 mV, SE=0.36; paired t-test, $t(31)=0.41$, $p=0.68$). Moreover, MEP amplitudes was equal before and after the RHI stimulation in both synchronous and asynchronous conditions, excluding a modulation of the overall cortical excitability due to the illusion or the course of the experiment (repeated measures ANOVA: interaction "temporal congruency X time": $F(1, 31)=0.074$, $p=0.79$) (Supplementary Figure 2).

SUPPLEMENTARY FIGURES and TABLES

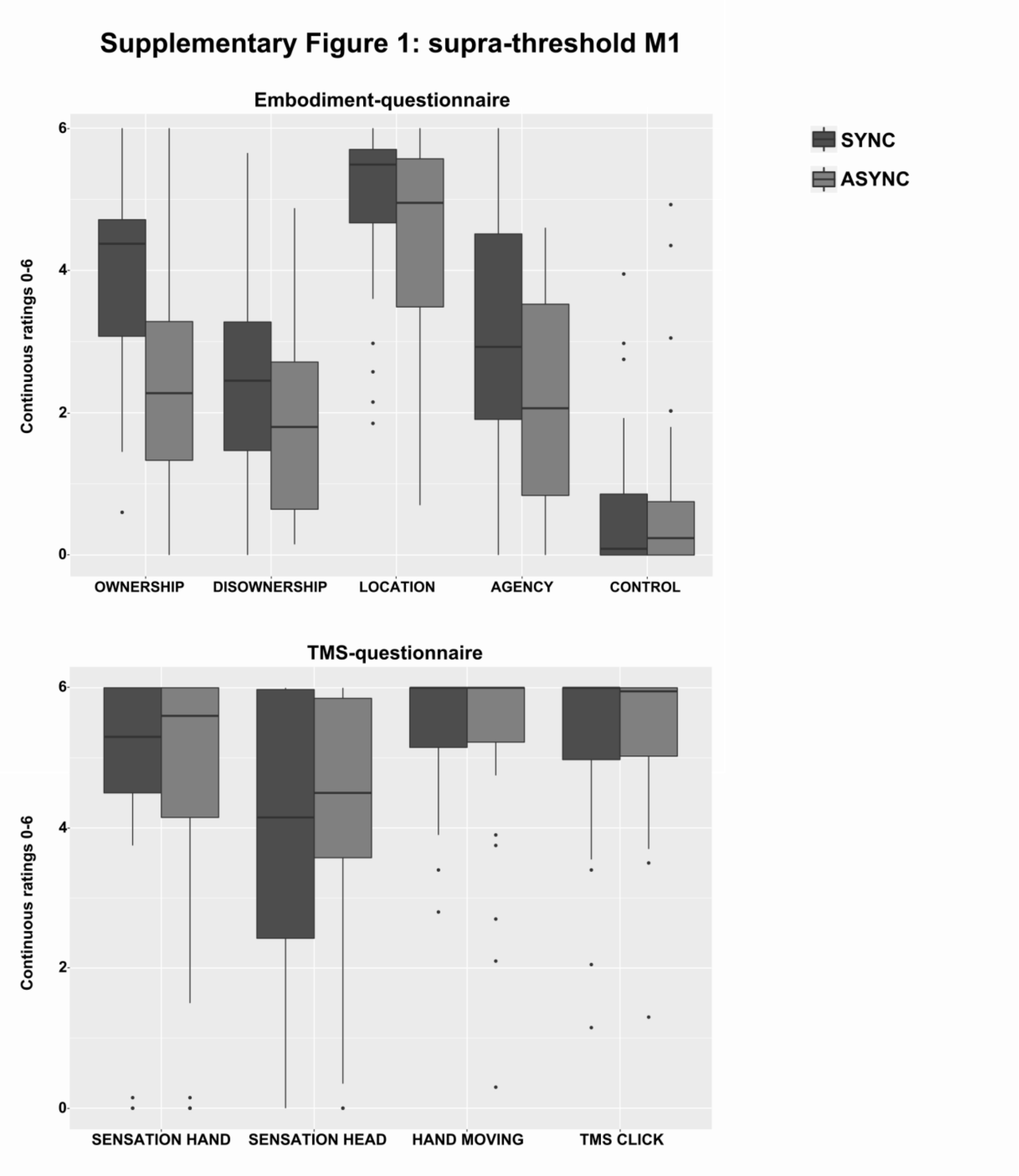

Supplementary Figure 1. Main experiment. Supra-threshold TMS over motor cortex (supra-threshold M1) time-locked with virtual reality feedback induces embodiment for a virtual hand
Figure shows results on subjective ratings on Embodiment-questionnaire (upper panel) and TMS-questionnaire (lower panel). "Boxes" are based on the first and third quartiles (inter-quartile range, lower and upper "hinges"), the median (line), the largest and the smallest value no further than 1.5 * the inter-quartile range (upper and lower whiskers), data beyond the end of the whiskers (points). Higher ratings (32 subjects) after synchronous (sync, dark grey) than (async, light grey) condition were reported for all embodiment statements, but not for the control questions (upper panel). Subjects' ratings on any sensation induced by TMS were comparable in synchronous and asynchronous conditions, excluding any generic effect of TMS between conditions (lower panel).

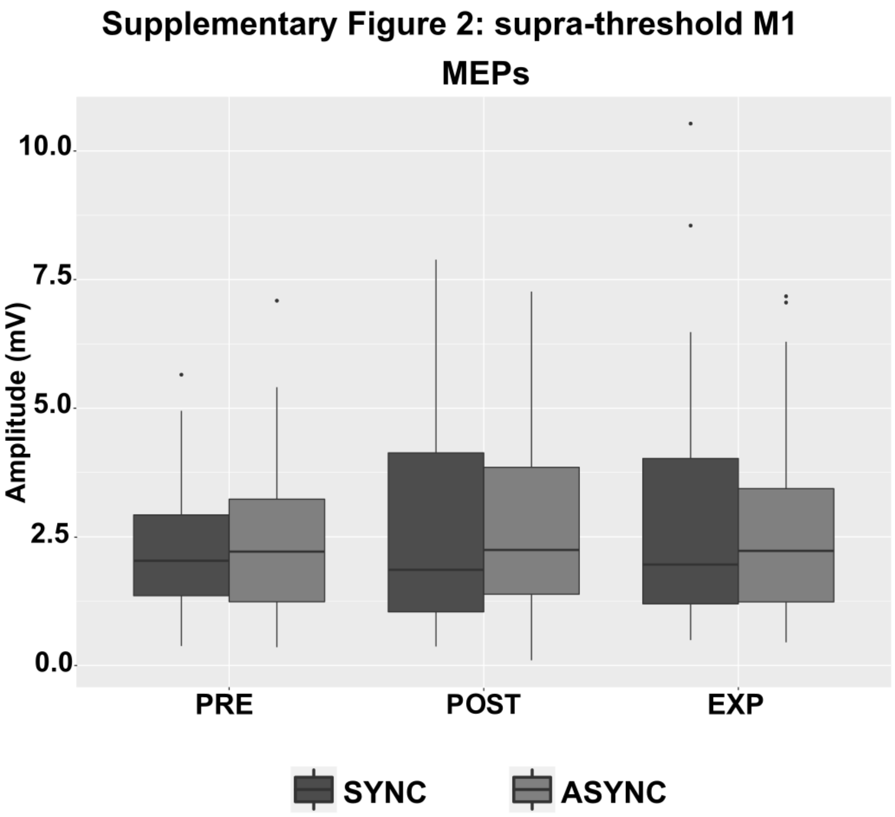

Supplementary Figure 2. Motor evoked potentials (MEPs) did not differ in synchronous or asynchronous condition
"Boxes" are based on the first and third quartiles (inter-quartile range, lower and upper "hinges"), the median (line), the largest and the smallest value no further than 1.5 * the inter-quartile range (upper and lower whiskers), data beyond the end of the whiskers (points). The amplitude of MEPs (32 subjects) was comparable in the synchronous (sync, dark grey) and asynchronous (async, light grey) condition when recorded before (PRE) and after (POST) TMS-VR stimulation (supra-threshold M1), as well as during the stimulation (EXP).

Supplementary Figure 3: MEPs

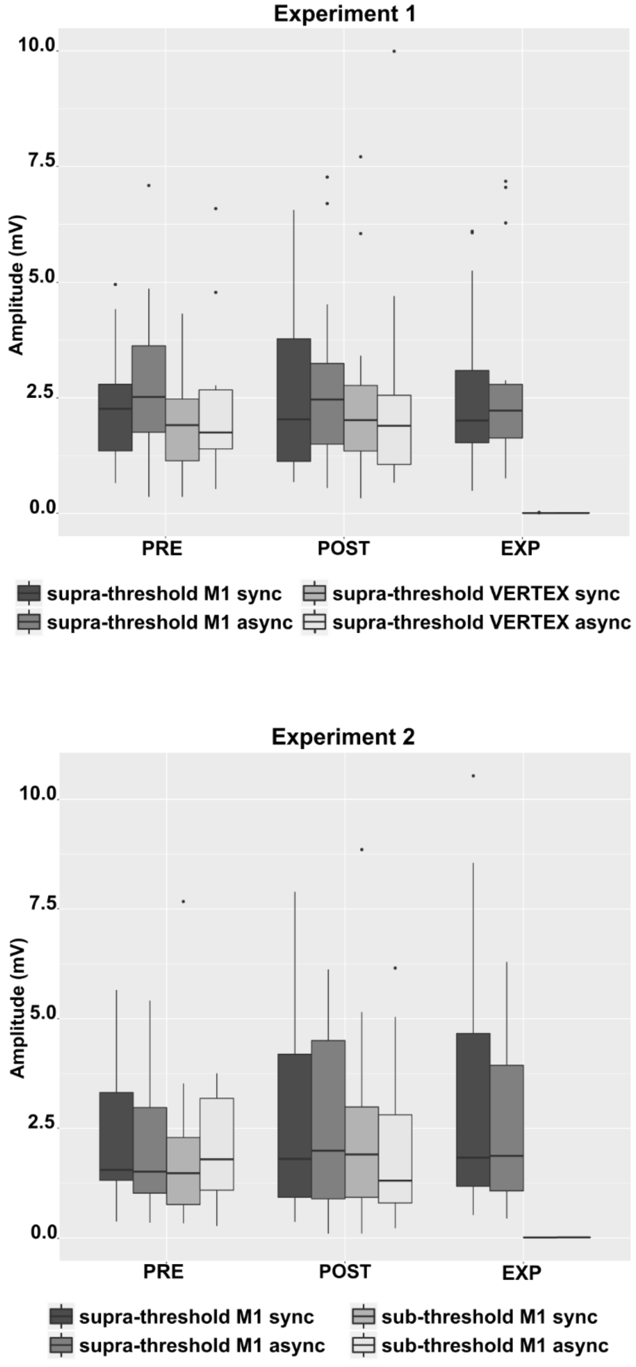

Supplementary Figure 3. Motor evoked potentials (MEPs) in experiment 1 and 2
Figure represents the amplitude of motor evoked potentials (MEPs) before (PRE), during (EXP) and after (POST) the TMS-VR stimulation in experiment 1 (supra-threshold M1 versus supra-threshold vertex, upper panel) and in experiment 2 (supra-threshold M1 versus sub-threshold M1, lower panel) (16 subjects). "Boxes" are based on the first and third quartiles (inter-quartile range, lower and upper "hinges"), the median (line), the largest and the smallest value no further than 1.5 * the inter-quartile range (upper and lower whiskers), data beyond the end of the whiskers (points). Differently from supra-threshold M1 stimulation, as expected, MEPs were absent during TMS-VR stimulation in supra-threshold vertex and in sub-threshold M1.

SUPPLEMENTARY TABLE 1: Experiment 1

Embodiment-questionnaire	OWNERSHIP	DISOWNERSHIP	LOCATION	AGENCY	CONTROL
supra-threshold M1 SYNC	3.72 ± 0.93	2.60 ± 0.65	4.83 ± 1.21	2.90 ± 0.73	0.45 ± 0.11
supra-threshold M1 ASYNC	2.13 ± 0.53	1.70 ± 0.43	4.11 ± 1.03	2.06 ± 0.52	0.37 ± 0.09
supra-threshold VERTEX SYNC	2.81 ± 0.70	2.30 ± 0.57	4.60 ± 1.15	2.49 ± 0.62	0.20 ± 0.05
supra-threshold VERTEX ASYNC	2.51 ± 0.63	2.34 ± 0.59	4.15 ± 1.04	2.51 ± 0.63	0.19 ± 0.05

TMS-questionnaire	HAND	HEAD	MOVEMENT	CLICK
supra-threshold M1 SYNC	4.47 ± 0.61	3.40 ± 0.61	5.63 ± 0.17	5.60 ± 0.28
supra-threshold M1 ASYNC	4.13 ± 0.67	3.67 ± 0.57	5.63 ± 0.16	5.58 ± 0.20
supra-threshold VERTEX SYNC	1.47 ± 0.52	3.97 ± 0.56	1.40 ± 0.48	5.38 ± 0.25
supra-threshold VERTEX ASYNC	1.48 ± 0.53	3.85 ± 0.65	1.58 ± 0.58	5.55 ± 0.25

Supplementary Table 1. Experiment 1 (supra-threshold M1 versus supra threshold vertex)

Means ± standard error of the ratings reported at the Embodiment-questionnaire (upper part) and TMS-questionnaire (lower part) in the experiment 1.

SUPPLEMENTARY TABLE 2: Experiment 2

Embodiment-questionnaire	OWNERSHIP	DISOWNERSHIP	LOCATION	AGENCY	CONTROL
supra-threshold M1 SYNC	4.16 ± 0.28	2.29 ± 0.38	5.08 ± 0.24	3.21 ± 0.44	0.80 ± 0.31
supra-threshold M1 ASYNC	2.49 ± 0.32	1.91 ± 0.26	4.49 ± 0.34	2.18 ± 0.39	1.17 ± 0.40
sub-threshold M1 SYNC	2.57 ± 0.39	2.16 ± 0.37	3.84 ± 0.41	2.31 ± 0.41	0.59 ± 0.19
sub-threshold M1 ASYNC	2.90 ± 0.33	1.58 ± 0.25	4.90 ± 0.18	2.62 ± 0.48	0.87 ± 0.27

TMS-questionnaire	HAND	HEAD	MOVEMENT	CLICK
supra-threshold M1 SYNC	5.16 ± 0.21	4.22 ± 0.39	5.27 ± 0.25	4.92 ± 0.35
supra-threshold M1 ASYNC	4.74 ± 0.44	4.55 ± 0.42	4.92 ± 0.44	5.06 ± 0.32
sub-threshold M1 SYNC	1.88 ± 0.50	3.51 ± 0.58	1.76 ± 0.48	4.52 ± 0.51
sub-threshold M1 ASYNC	1.79 ± 0.53	3.40 ± 0.52	1.58 ± 0.47	4.83 ± 0.45

Supplementary Table 2. Experiment 2 (supra-threshold M1 versus sub-threshold M1)

Means ± standard error of the ratings reported at the Embodiment-questionnaire (upper part) and TMS-questionnaire (lower part) in the experiment 2.
