# Supplementary material for: Non‐invasive brain stimulation of motor cortex induces embodiment when integrated with virtual reality feedback
Source: Eur J Neurosci. 2018 Mar 9;47(7):790–9. doi: 10.1111/ejn.13871 (PMC5900900; doi:10.1111/ejn.13871)
Supplement: Supplementary file 1 [file EJN-47-790-s001.pdf]

EJN European Journal of Neuroscience FENS Federation of European Neuroscience Societies

Non-invasive brain stimulation of motor cortex induces embodiment when integrated with virtual reality feedback

Bassolino M., Franza M., Bello Ruiz J., Pinardi M., Schmidlin T., Stephan M.A., Solca M., Serino A. & Blanke O.

Review timeline:

Submission date:	03 August 2017
Editorial Decision:	09 October 2017
Revision received:	13 December 2017
Editorial Decision:	08 January 2018
Revision received:	15 January 2018
Editorial Decision:	17 January 2018
Revision received:	09 February 2018
Accepted:	09 February 2018

Editor: Gregor Thut

1st Editorial Decision 09 October 2017

Dear Dr. Bassolino,

Your manuscript has now been seen by two expert reviewers and one of our senior section editors. Based on their comments, we regret that we cannot publish your manuscript in EJN in its current form. However, the research described in your manuscript is of interest and novel and therefore we invite you to resubmit a much revised version.

As you will see, both reviewers commend your manuscript (for its technical achievements, interesting and novel results) but also raise important concerns. These include concerns about writing and data presentation (long but inconsistent data reporting, reviewer 1), an uncommon experimental design (reviewer 1) and caveats to the interpretation because of the lack of a crucial control condition (reviewer 2) alongside more minor points (mostly clarifications, specifications, “simplifications” and typos).

Thank you for submitting your work to EJN.

Kind regards,

Paul Bolam & John Foxe
co-Editors in Chief, EJN

Reviews:

Reviewer: 1 (Nicholas Holmes, University of Nottingham, UK)

Comments to the Author
Summary
Bassolino and colleagues conducted an experiment with 32 participants, all of whom performed the main experimental condition, but half of whom performed one kind of control experiment, and the other half performed a different control experiment. The main aim was to evoke the well-known and much-studied 'rubber (or virtual) hand illusion' (RHI), not by physically touching or moving the participant's hand, but by inducing movements by applying TMS over the motor cortex, and yoking these to VR-generated movements of a hand. They succeed in this task - ratings of the RHI (questionnaires) revealed stronger agreements with the 'illusion' questions than the 'control'

questions, and stronger agreement with the questions when the visual stimulus moved after a short delay ('synchronous') rather than a long and variable delay ('asynchronous').

Evaluation
Technically, the results of the manuscript are an achievement - to yoke the VR and TMS systems with a relatively small delay (~65ms). The study does not, however, advance our understanding of the rubber hand illusion or the physiology of the motor control system, so this report should be seen as a solid methodoligical achievement. Hopefully the authors will develop this method further.

My main concerns are first with the writing - the manuscript is very long, there are very many results, graphs, and tables, but the statistics and results are selectively and inconsistently reported. Please see all the comments below. My main suggestion here would be to simplify the manuscript and the writing so that the results are easy to follow. The statistical tests should test the manuscript's hypotheses, rather than just be reported in full. For the RHI questions with N=32, each question is analysed separately, with bonferroni corrections, then the full interaction term of an ANOVA is given; but, for control conditions, the interaction between synchrony and question seems to be missing - why?

Second, the experimental design is strange, with two different groups doing two different control experiments, and I would encourage the authors to report them first as two separate experiments, then only as a supplementary analysis to examine the pooled effects. For example, with 32 participants, the RHI data show significant differences between all 4 components of the illusions (Figure 2, upper panel), but with the two groups of 16 participants, these differences do not pass the significance criterion (Figure 4, upper panel, first two questions show differences; Figure 5, upper panel, first and 4th questions show differences). Clearly, the questionnaire data are underpowered with N=16, leading to non-significant contrasts and, apparently, to no significant interaction between synchrony and question. Combining these two well-controlled, but individually underpowered experiments into one main experiment and two sub-experiments, which are seemingly reported as having been run at the same time, but in two counterbalanced sessions a week apart, is very confusing. I suspect two experiments were run, but that the results look better when combined. If that's what happened, that's what the authors should report.

Minor
Page 5
- specify what the reported errors are - SE, SD?
- change 'exclude that' to 'test the alternative hypothesis that' - experiments should be run to test (alternative) hypotheses, not to 'exclude' unwanted explanations - the authors should be open to being wrong!
- 'TMS stimulation on M1' - this should be 'TMS over M1', correct throughout
- 'Declaration of Helsinki' which version? the latest version requires pre-registration of all studies with humans

6
'drift' - should this be 'DRIFT'?

7
- were the 'main experimental condition' and 'additional condition' run in counterbalanced order? not quite clear from the first paragraph here

8
- '70mm' -is that outer diameter, inner diameter?
- '...due to the depth of the cortex...' - this is likely not true. (My own measurements of the distance between vertex and cortex, in a group of 20 MRI scans, show that the vertex is, on average, 17.7mm from the cortex, while the cortex under C3/C4, the approximate location of M1, is 14.4mm. 3.3mm is not a large difference.) If the authors want to claim that vertex TMS at 130% of RMT does not stimulate the brain, they need to present evidence, measure the distance on any available MRI scans they have, and do the correcton for distance suggested by Stokes MG, Chambers CD, Gould IC, English T, McNaught E, McDonald O, Mattingley JB (2007) Distance-adjusted motor threshold for transcranial magnetic stimulation. Clinical Neurophysiology, 118(7):1617-1625.
- '12.2 ms' - change to 's'!

9
- '...(>0.05 mV) preceding the TMS pulse were excluded from analysis.' - specify exactly: how long was the window, how many time-points had to be >.05; is this peak-to-peak? did the authors use RMS or other processing to determine this?
- 'acceleration modulus' - please explain to non-mathematicians, is this just 'absolute' or 'unsigned'?

10
- the abbreviations SYNC and ASYNC are defined multiple times. Either delete them all, or define them once and use consistently

11
- 'in healthy young subjects the latency of muscle twitches in the FDI muscle is usually around the 20 ms (Groppa et al., 2012) while the onset of evoked hand movements is usually between 15 and 55 ms after the TMS pulse (Finisguerra et al., 2015).' - how is it possible that the finger moves before (15ms) the muscle that moves it is activated (20ms)? something is wrong here. If the kinematic data were filtered, then it could appear that the movement began earlier than 20ms, but it *must* be impossible for pre-muscle-movement to occur, right? Or is the early finger movement caused by activation of unrecorded muscles?
- why should there be any proprioceptive drift? What was the difference in distance and direction of the real hand and the virtual hand? Explain before introducing the measurement that is taken from the hand positions

13
- 'TMS stimulations' - the 'S' already means 'stimulation'; please remove extra words, change to 'conditions'?
- TMS over M1 and TMS over vertex feel different - what is the evidence that they should feel the same 'general sensations' for the two sites?

15
- '(paragraph 1)' - typo?
- most of this paragraph repeats the methods, as if it was prepared for another journal - cut out the repetition please
- 'SYNC SUPRA-T-M1 versus ASYNC' - the number of capitalised acronyms in this report makes reading very difficult. I am sure the authors can find a nicer, simpler way to name the relatively few experimental conditions
- 'Bonferroni correction: alpha set at 0.01, 0.05/5, dz: 0.98; Cohen's d value, calculated as within-subjects effect size using G Power matched pairs statistical tests' - I don't understand any of this. Why are there 5 comparisons to be corrected? Why is Cohen's d being calculated with G power rather than by, e.g., dividing the t-statistic by sqrt(N)? Please re-write the code in brackets here in full sentences, or remove if not needed)
- 'main interaction, temporal congruency, SYNC versus ASYNC X 5 components' - should this be 'interaction between congruence and question'? please simplify the reporting

16
- the second paragraph is too complex, and does not make sense. 'No difference...' - between what and what? SYNC and ASYNC?
- please report actual p-values, not, e.g., 'p>.013' or 'F(3, 90)=2.34, p>.05)'. P-values should be exact. The readers need to be able to make their own minds up about the data. The authors are using the '>' symbol to hide how close the p-values are to 0.05. on page 17, for example, p is reported as p=.79 when it favours the authors' interpretation
- 'alpha set at 0.05/4 = 0.025' - what is wrong here? Same above: 0.013 should be 0.0125. If p-values are going to be interpreted according to a cliff-edge (> or <), then it is critical that the alpha is not rounded up or down
- 'a positive drift' - give the means and SD/SE of drift in all relevant conditions
- '>90%' - give SD/SE with all means. All t-tests should be accompanied with means and SD/SE for the relevant conditions. p-values tell us very little of interest on their own

17
- 'V' - please use words wherever possible. 'vertex' is much easier to read and follow than 'V'

- 'M1: p=.044.; V: p=0.89' - please report p-values consistently: with or without leading 0?
- 'congruent visual feedback' - how is the feedback 'congruent' if no movement was evoked by TMS?

18
- 'without any differences between' - give means, and statistics that allow these conclusions to be drawn
- 'were equally present' - yes, OK, but I expect that any rating of sensation *intensity* will be stronger for M1 than V conditions
- 'confirming that participants perceived M1 and V conditions in a similar way' - no, the TMS questions asked only if participants 'Perceived anything...' - they did not ask about the quality or quantity of those perceptions. All the authors can say, I think, is that the TMS conditions produced similar *numbers* of perceptions, not 'similar' perceptions.

20
- 'only 1 movement induced out of 36 delivered pulses in 1 a single subject in the SYNC condition and in another subject in the ASYNC condition' - please re-write this

Reviewer: 2 (Francesca Garbarini, University of Turin, Italy)

Comments to the Author
In the present study, Bassolino et al. induced a virtual hand embodiment by combining Transcranial Magnetic Stimulation (TMS), to stimulate the hand primary motor cortex (M1), and Virtual Reality (VR), to contrast synchronous and asynchronous visual feedbacks, mimicking the involuntary hand twitches induced by the TMS. They found that, when the TMS pulses were delivered above the resting motor threshold (rMT) and were able to induce hand twitches, the temporal coherence between the TMS pulses and the observed virtual hand twitches induced the embodiment of the virtual hand. In particular, the above rMT procedure was able to replicate the significant difference between synchronous and asynchronous conditions, as measured by subjective questionnaires. No difference between synchronous and asynchronous conditions was found for the proprioceptive drift. On the contrary, when (in a control experiment) the TMS pulses were delivered under the rMT and were not able to induce hand twitches, the temporal coherence between the TMS pulses and the observed virtual hand twitches did not induce the embodiment.

This is a very interesting study, including well-designed main and control experiments and providing clear and novel results in the field. However, I only have a major concern that the authors can take into account for their revision.

My major concern is related to the results interpretation that, in my view, need to be supported by a crucial control experiment. According to the authors, the obtained illusory embodiment is explained by the central stimulation of the hand motor representation in the brain. They compared their results to the Collins et al. 2017's results, showing that invasive stimulation of the primary somatosensory cortex was able to induce the embodiment when synchronous visual stimuli were delivered to the fake hand (and no tactile stimulation was delivered to the real hand). However, a more conservative interpretation can explain their results as a passive movement effect, well known in the motor version of the rubber hand illusion. Indeed, only when involuntary hand twitches were induced by the supra rMT TMS the illusion occurred. Involuntary hand twitches, induced by TMS, can be considered as passive, externally triggered, involuntary hand movements. The subjects are usually aware of the hand twitches, they are able to feel them and to report if they occur or not (see for instance, Bruno et al., The role of premotor and parietal cortex during monitoring of involuntary movement: a combined TMS and tDCS study, Cortex 2017). Accordingly, in the present experiment, the TMSq results show that the subjects gave greater ratings for hand movement sensations during SUPRA-T-M1 condition (the one able to induce hand twitches). Thus, even if the authors try to explain away this interpretation suggesting that hand twitches are smaller and shorter than passive movements (I actually do not understand very well the second argument related to the contact between the device and the hand), I think that a control experiment is necessary to support their interpretation. Thus, I strongly suggest to include a control experiment in which hand twitches are induced by a peripheral stimulation. According to the authors' interpretation, we can expect that a peripheral stimulation is not able to induce the virtual hand illusion. They can evaluate different procedures to peripherally induce hand twitches; maybe, they can consider stimulating the median nerve at the wrist. If crucial

EJN European Journal of Neuroscience FENS Federation of European Neuroscience Societies

difference will be found between central (TMS over M1) and peripheral stimulations, they can prove that their results cannot be explained as a passive movement effect but they represent the effect of the central stimulation of the hand motor representation in the brain.

Authors' Response 13 December 2017

Reviewer: 1

Comments to the Author
Summary
Bassolino and colleagues conducted an experiment with 32 participants, all of whom performed the main experimental condition, but half of whom performed one kind of control experiment, and the other half performed a different control experiment. The main aim was to evoke the well-known and much-studied 'rubber (or virtual) hand illusion' (RHI), not by physically touching or moving the participant's hand, but by inducing movements by applying TMS over the motor cortex, and yoking these to VR-generated movements of a hand. They succeed in this task - ratings of the RHI (questionnaires) revealed stronger agreements with the 'illusion' questions than the 'control' questions, and stronger agreement with the questions when the visual stimulus moved after a short delay ('synchronous') rather than a long and variable delay ('asynchronous').
Evaluation
Technically, the results of the manuscript are an achievement - to yoke the VR and TMS systems with a relatively small delay (~65ms). The study does not, however, advance our understanding of the rubber hand illusion or the physiology of the motor control system, so this report should be seen as a solid methodoligical achievement. Hopefully the authors will develop this method further.

1. **My main concerns are first with the writing - the manuscript is very long, there are very many results, graphs, and tables, but the statistics and results are selectively and inconsistently reported. Please see all the comments below. My main suggestion here would be to simplify the manuscript and the writing so that the results are easy to follow. The statistical tests should test the manuscript's hypotheses, rather than just be reported in full.**

We thank the Reviewer for his/her evaluation and suggestions. The Results have now been re-written following the Reviewer's suggestion by firstly showing the two experiments separately. The analysis related to the 32 participants and the related figures (figure 2 and 3 in the previous version) have been moved to the Supporting Information (now Supplementary Figure 1 and 2). The same was done for the figures representing the results related to MEPs and TMS-evoked-movements (figure 6 in the previous version, now Supplementary figure 3) and for the new version of Table 1 and 2 reporting means and standard errors of the results related to Embodiment-questionnaire and Tms-questionnaire in Experiment 1 and 2 . The Results session has been significantly shortened (from 1541 to 1200 words).

2. **For the RHI questions with N=32, each question is analysed separately, with bonferroni corrections, then the full interaction term of an ANOVA is given; but, for control conditions, the interaction between synchrony and question seems to be missing - why?**

We understand the Reviewer's concern. To compare for multiple comparisons with the same approach in all the experiments, we have now provided new statistical analyses.
We realized that given the unbalanced number of components related to the embodiment statements (4 components) and related to the control item (1 component) in the Embodiment questionnaire, it is more appropriate to perform two separate repeated measures ANOVAs respectively on the 4 components related to embodiment (ownership, agency, location, disownership for the physical hand) and on the control component (Longo et al., 2008). This approach allows us to test any possible difference among ownership, agency, location, disownership and to properly control for response biases with the control component. Combining those analyses into an unique ANOVA would mask specific embodiment versus control differences.
The new analysis confirms the previous results: supra-threshold M1 stimulation induces illusory feeling of ownership toward the virtual hand when combined with temporally congruent visual feedback of the virtual hand moving accordingly with the TMS-evoked movements of the physical hand.

EJN European Journal of Neuroscience FENS Federation of European Neuroscience Societies

A new paragraph describing the statistical analysis has been added (pages 15-16). Results have been re-written.

3. **Second, the experimental design is strange, with two different groups doing two different control experiments, and I would encourage the authors to report them first as two separate experiments, then only as a supplementary analysis to examine the pooled effects. For example, with 32 participants, the RHI data show significant differences between all 4 components of the illusions (Figure 2, upper panel), but with the two groups of 16 participants, these differences do not pass the significance criterion (Figure 4, upper panel, first two questions show differences; Figure 5, upper panel, first and 4th questions show differences). Clearly, the questionnaire data are underpowered with N=16, leading to non-significant contrasts and, apparently, to no significant interaction between synchrony and question. Combining these two well-controlled, but individually underpowered experiments into one main experiment and two sub-experiments, which are seemingly reported as having been run at the same time, but in two counterbalanced sessions a week apart, is very confusing. I suspect two experiments were run, but that the results look better when combined. If that's what happened, that's what the authors should report.**

It is true that the crucial comparison between supra-threshold motor cortex stimulation and the control conditions of sub-threshold and non-motor (vertex) stimulation are described in experiments 1 and 2, while the analysis including all the 32 subjects is an additional analysis. Thus, following the Reviewer's suggestion, the two experiments are now reported in the revised version of the manuscript first as separate experiments 1 and 2. Then, in the Supplementary information we also report them as a combined analysis of the 32 subjects. This has also been changed in the revised version of methods and in the results.

In both experiment 1 and 2, in the illusion condition (supra-threshold motor cortex stimulation), there is a significant synchronous-asynchronous difference in the ratings to the ownership component, our main component of interest among the 4 studied "embodiment components" that also included agency, location, and disownership for the real hand. The new analysis including the factor "embodiment" (4 levels) tests any possible difference among ownership and the other embodiment components.
The sample size (n=16) was decided on the basis of a power analysis calculated on the ratings to our component of interest (ownership) collected in a pilot experiment (N=4) conducted to test the set-up (e.g. delays, duration). This pilot analysis showed that by collecting data in 16 subjects we should be able to find a synchronous-asynchronous difference for the ownership component. This sample size estimation was also confirmed in the "a posteriori" power analysis on the 32 subjects and reported in the previous version of the manuscript. The analysis conducted in the 32 subjects show that the illusion could induce further effect in other components of the embodiment, only when the sample size is increased, thus demonstrating that illusory feeling of agency, changes in location and disownership of the real hand are less strong than the effect in ownership component.

The related part in the revised discussion has been now changed (page 23):
The present supra-threshold M1 stimulation when combined with temporally congruent virtual feedback induced an illusory feeling of ownership towards the virtual hand (experiment 1 and 2). Illusory embodiment involved all main components of hand embodiment as described for the RHI induced by manual stimulation protocols (Longo et al., 2008) (ownership, agency, location, disownership for the physical hand) when a larger sample size is considered (see the analysis in 32 subjects, Supporting information).

The fact that our main component of interest was related to the "ownership for the virtual hand" has now been specified also in the Method (page 12):

The items of the Embodiment-questionnaire were selected from those used previously to test different embodiment components (Longo et al., 2008). Our main component of interest was related to ownership for the virtual hand ("it seemed like the virtual hand was part of my body"; "it seemed like the virtual hand was my hand"). Considering previously reported dissociations between ownership and other embodiment-related components (e.g. Longo et al., 2008; Serino et al., 2013) we also included in the questionnaire the following additional components: disownership for the physical hand ("it seemed like my hand had disappeared"; "it seemed like the experience on my hand was less vivid than normal");location ("it seemed like the virtual hand was in the location where my hand was"; "it seemed like my hand was in the location where the virtual

hand was") and agency ("it seemed like I was in control of the virtual hand"; "it seemed like I could have moved the virtual hand if I had wanted").

Minor
Page 5
- specify what the reported errors are - SE, SD?
We reported standard deviation. This has been now specified in the revised text.

- change 'exclude that' to 'test the alternative hypothesis that' - experiments should be run to test (alternative) hypotheses, not to 'exclude' unwanted explanations - the authors should be open to being wrong!
This part has been changed (page 5):

A first group of 16 subjects (mean age 29.8 years, SD ± 4.8, 8 females) underwent the "vertex" control condition (supra-threshold vertex), to test the alternative hypothesis that unspecific TMS effects unrelated to the activation of the M1 could induce embodiment for the virtual hand (experiment 1)*.*

- 'TMS stimulation on M1' - this should be 'TMS over M1', correct throughout
This has been corrected.

- 'Declaration of Helsinki' which version? the latest version requires pre-registration of all studies with humans
Thank you for this suggestion. We have removed this part.

6
'drift' - should this be 'DRIFT'?
We have corrected this in the manuscript: for simplicity, in the new version of the manuscript "DRIFT" has been always changed to "drift".

7
- were the 'main experimental condition' and 'additional condition' run in counterbalanced order? not quite clear from the first paragraph here
This has been now explicitly written (page 7):

The subjects performed the main experimental condition (supra-threshold M1) on one day and one additional condition (either supra-threshold vertex or sub-threshold M1, depending on the group) on a different day, in a counterbalanced order*, and with an interval of approximately 1 week between the two testing days.*

8
- '70mm' -is that outer diameter, inner diameter?
We referred to the diameter of the wing. This has be added in the text.

- '...due to the depth of the cortex...' - this is likely not true. (My own measurements of the distance between vertex and cortex, in a group of 20 MRI scans, show that the vertex is, on average, 17.7mm from the cortex, while the cortex under C3/C4, the approximate location of M1, is 14.4mm. 3.3mm is not a large difference.) If the authors want to claim that vertex TMS at 130% of RMT does not stimulate the brain, they need to present evidence, measure the distance on any available MRI scans they have, and do the correcton for distance suggested by Stokes MG, Chambers CD, Gould IC, English T, McNaught E, McDonald O, Mattingley JB (2007) Distance-adjusted motor threshold for transcranial magnetic stimulation. Clinical Neurophysiology, 118(7):1617-1625.
We recognize that this citation is misleading. We have removed this part from the text.

- '12.2 ms' - change to 's'!
We thank the Reviewer for this observation. We have corrected this typo in the text.

9

- '...(>0.05 mV) preceding the TMS pulse were excluded from analysis.' - specify exactly: how long was the window, how many time-points had to be >.05; is this peak-to-peak? did the authors use RMS or other processing to determine this?
We considered the peak-to-peak amplitude in a time window of 100 ms preceding the TMS pulse. This has been specified in the text (page 9):

Trials with EMG background activity (peak-to-peak amplitude>0.05 mV in the 100 ms preceding the TMS pulse) were excluded from analysis.

- 'acceleration modulus' - please explain to non-mathematicians, is this just 'absolute' or 'unsigned'?
The modulus of the acceleration is calculated as the square root of the sum of the squares of the components (i.e. the three axes) (this is a direct result of the Pythagorean theorem). This has been specified in the text (page 9).

10
- the abbreviations SYNC and ASYNC are defined multiple times. Either delete them all, or define them once and use consistently
Thank you for this suggestion. According to requirement to reduce abbreviations, we have now used the terms synchronous–asynchronous in the main text and the related abbreviations only in the figure as specified in the new figure legend (e.g. Figure 2 and 3).

11
- 'in healthy young subjects the latency of muscle twitches in the FDI muscle is usually around the 20 ms (Groppa et al., 2012) while the onset of evoked hand movements is usually between 15 and 55 ms after the TMS pulse (Finisguerra et al., 2015).' - how is it possible that the finger moves before (15ms) the muscle that moves it is activated (20ms)? something is wrong here. If the kinematic data were filtered, then it could appear that the movement began earlier than 20ms, but it *must* be impossible for pre-muscle-movement to occur, right? Or is the early finger movement caused by activation of unrecorded muscles?
We understand the Reviewer's concern. The time window indicated for the movements (15-55 ms after the TMS pulse) were chosen to include any possible cases in which a latency of movement onset or MEPs slightly inferior of the mean estimation of 20 ms (e.g. 18 or 19 ms) could occur. However, this did not happen (this has been verified by performing again the analysis by considering a 20-55 ms time window). So, we have now substitute the previous reported "15 ms" with "20 ms" in the text.

- why should there be any proprioceptive drift? What was the difference in distance and direction of the real hand and the virtual hand? Explain before introducing the measurement that is taken from the hand positions
Following the Reviewer's suggestion, in the new version the explanation related to the displacement of the virtual hand has been already introduced in the paragraph about VR (page 10):

During the TMS-VR stimulation, the virtual hand was displaced leftward with respect to the perceived position of the real hand as defined in an initial calibration phase.

In addition, this aspect has been also recalled in the paragraph about the proprioceptive drift (page 13):

Proprioceptive drift. The hand location task (proprioceptive drift) was similar to that described elsewhere (Tsakiris 2010 for a review). Before and after the TMS-VR stimulation, participants were asked to indicate the perceived position of their real right hand in VR by using a keyboard with their left hand to move a cursor on a white empty table. Given that during the TMS-VR stimulation the virtual hand was displaced with respect to the perceived position of participants' real hand, we verified if after the stimulation participants reported the perceived position of their hand as drifted toward the location of the virtual hand.

13
- 'TMS stimulations' - the 'S' already means 'stimulation'; please remove extra words, change to 'conditions'?

EJN European Journal of Neuroscience FENS Federation of European Neuroscience Societies

Following the Reviewer's suggestion, the term "stimulation" after TMS has been removed and it has been substituted with the term "condition" when appropriate (e.g. in the paragraph "Control measures": In order to assess our subjects' perception to the different TMS conditions (supra-threshold M1, supra-threshold VERTEX, sub-threshold M1), participants responded also to a *TMS questionnaire* ….)

- TMS over M1 and TMS over vertex feel different - what is the evidence that they should feel the same 'general sensations' for the two sites?

TMS over M1 and TMS over vertex (or sub-threshold) could induced similar sensations because of the contact between the coil and the scalp and because of the sound clicks. For these reasons, we tested if participants perceived similar sensations related to the head ("head sensation" in the TMS-questionnaire) and to the sound clicks (question related to the sound) in both conditions. It is worth noticing that as during synchronous supra-threshold stimulation, even during vertex stimulation or sub-threshold stimulation, both head sensation and sound clicks were temporally congruent with the movement of the virtual hand, so that in principle these aspects could have induced the illusion. However, our previous analysis as well as the new analysis showed that this is not the case. More details have been added now in the text (page 13):

The general sensations induced by TMS are expected to be similar in all the conditions, because of the contact between the coil and the scalp and because of the TMS sound clicks. In contrast, the sensations regarding the hand are specific to the supra-threshold M1 condition.

This has been also directly tested in the new analysis related to the TMS questionnaire that includes the additional 4 levels within-subjects factor "sensations". Results show that subjects perceived differently the sensations on the hand (hand sensation and perception of TMS-evoked hand movements) during supra-threshold M1 and supra-threshold VERTEX or sub-threshold M1 stimulation, despite similar general effects of TMS related to the contact with the scalp or the sound click in all conditions.

15

- '(paragraph 1)' - typo?

- most of this paragraph repeats the methods, as if it was prepared for another journal - cut out the repetition please

This paragraph has been removed (including the typo). The statistical information previously included in that part of the text has been now inserted in a new additional paragraph titled "Statistical analysis" introduced before the Result section (page 15):

Statistical analysis. A repeated measures ANOVA were run on subjective ratings of the four components related to the embodiment (ownership, disownership, location, agency) with "embodiment components" (4 levels), "temporal congruency" (synchronous versus asynchronous) and "site" (experiment 1, M1 versus vertex) or "intensity" (experiment 2, supra-threshold versus sub-threshold) of stimulation as within-subjects factors. Similar repeated measures ANOVAs with the within-subjects factors "temporal congruency" and "site" (experiment 1) or "intensity" (experiment 2) of stimulation were performed on (i) the ratings related to the control component of the Embodiment-questionnaire; (ii) on the perceived position of participants' hand reported in the proprioceptive drift task (difference between post and pre); (iii) on ratings to the 4 items of the TMS-questionnaire with the additional within-subjects factor "sensations" (4 levels: sensations related to the hand, to the head, to the TMS-evoked movements and to the sound clicks); (iv) on the MEPs amplitude before and after the stimulation with the supplementary within-subject factor of "time" (2 levels: pre versus post). If the ANOVA revealed a significant interaction, we correct for multiple comparisons by using Newman-Keuls post hoc tests. The percentage of TMS-evoked movements and the MEP amplitude during the stimulation were compared in synchronous versus asynchronous condition by means of paired t-tests, Bonferroni corrected. An additional analysis about the supra-threshold M1 stimulation putting together data from experiment 1 and from experiment 2, has been included in Supplementary Information (see also supplementary figure 1 and 2).

- 'SYNC SUPRA-T-M1 versus ASYNC' - the number of capitalised acronyms in this report makes reading very difficult. I am sure the authors can find a nicer, simpler way to name the relatively few experimental conditions

For the revised manuscript, we decided to keep the capitalized acronyms of M1, while vertex and supra/ sub threshold are written in full, as well as synchronous-asynchronous condition. So the conditions are synchronous-asynchronous supra-threshold M1, sub-threshold M1, supra-threshold vertex. Other not

necessary abbreviations have also been now removed (TMS-questionnaire, Embodiment-questionnaire, repeated measures ANOVA, resting motor threshold, head mounted display). To assure readability, differently for all the other changes in the text, the modifications related to the abbreviations are not marked in red.

- 'Bonferroni correction: alpha set at 0.01, 0.05/5, dz: 0.98; Cohen's d value, calculated as within-subjects effect size using G Power matched pairs statistical tests' - I don't understand any of this. Why are there 5 comparisons to be corrected? Why is Cohen's d being calculated with G power rather than by, e.g., dividing the t-statistic by sqrt(N)? Please re-write the code in brackets here in full sentences, or remove if not needed)
This part was clarified and moved to Supplementary Information (see the comment above).
A new analysis has been performed including the additional factor “embodiment components”.
We recognize that the calculation of the effect size (as well as the reference to the software used to calculate Cohen’s d value) is not necessary here for this additional analysis and we removed it.

- 'main interaction, temporal congruency, SYNC versus ASYNC X 5 components' - should this be 'interaction between congruence and question'? please simplify the reporting
This part has been removed (see above a previous comment of the same Reviewer).

16
- the second paragraph is too complex, and does not make sense. 'No difference...' - between what and what? SYNC and ASYNC?
- please report actual p-values, not, e.g., 'p>.013' or 'F(3, 90)=2.34, p>.05)'. P-values should be exact. The readers need to be able to make their own minds up about the data. The authors are using the '>' symbol to hide how close the p-values are to 0.05. on page 17, for example, p is reported as p=.79 when it favours the authors' interpretation
- 'alpha set at 0.05/4 = 0.025' - what is wrong here? Same above: 0.013 should be 0.0125. If p-values are going to be interpreted according to a cliff-edge (> or <), then it is critical that the alpha is not rounded up or down

This part refers to the analysis of the 32 subjects and it has been now moved to Supplementary information. A new analysis has been performed including the additional factor “embodiment components” in the ANOVA. The paragraph has been re-written and modified by reporting p values in full.

- 'a positive drift' - give the means and SD/SE of drift in all relevant conditions
This has been added in the text.

- '>90%' - give SD/SE with all means. All t-tests should be accompanied with means and SD/SE for the relevant conditions. p-values tell us very little of interest on their own
This has been specified in the text**.**

17
- 'V' - please use words wherever possible. 'vertex' is much easier to read and follow than 'V'
“V” has been substituted by “vertex”.

- 'M1: p=.044.; V: p=0.89' - please report p-values consistently: with or without leading 0?
In the new version of the manuscript, we have always reported p-values with leading 0.

- 'congruent visual feedback' - how is the feedback 'congruent' if no movement was evoked by TMS?
We meant that the visual feedback of the moving hand in VR was synchronous with the vertex stimulation. However, we understand the possible confound and we have removed the sentence in the new version of the results.

18
- 'without any differences between' - give means, and statistics that allow these conclusions to be drawn
Means± SE and statistics are fully reported in Table 1 of Supplementary Information. We now refer to Table 1 at this point in the text in the new version of the manuscript.

- 'were equally present' - yes, OK, but I expect that any rating of sensation *intensity* will be stronger for M1 than V conditions
We understand the Reviewer's comment. However, this was not the case for the sensation related to the head or the TMS sound clicks, while it was indeed true for hand-related perceptions (please see the results of experiment 1, page 17). In fact, participants rated very similarly head sensations and the TMS sound clicks in both M1 and vertex conditions. In both cases, the same intensity of stimulation was applied, thus it is possible that the different coil positions did not significantly influenced neither the perception of the TMS sound clicks neither a possible sensations on the scalp.

- 'confirming that participants perceived M1 and V conditions in a similar way' - no, the TMS questions asked only if participants 'Perceived anything...' - they did not ask about the quality or quantity of those perceptions. All the authors can say, I think, is that the TMS conditions produced similar *numbers* of perceptions, not 'similar' perceptions.
We understand the Reviewer's suggestion. The sentence has been changed (page 17):
Altogether these data suggests that participants gave different ratings after M1 and vertex stimulation related to the hand-effects evoked by the TMS (sensation and movement), but similar scores to the general TMS effects, such as the TMS-induced somatosensory sensation on the head and the TMS sound clicks.

20
- 'only 1 movement induced out of 36 delivered pulses in 1 a single subject in the SYNC condition and in another subject in the ASYNC condition' - please re-write this
This part has been modified (page 20):
1 movement out of 36 delivered TMS pulses in two subjects, in synchronous and in asynchronous condition, respectively.

Reviewer: 2

Comments to the Author
In the present study, Bassolino et al. induced a virtual hand embodiment by combining Transcranial Magnetic Stimulation (TMS), to stimulate the hand primary motor cortex (M1), and Virtual Reality (VR), to contrast synchronous and asynchronous visual feedbacks, mimicking the involuntary hand twitches induced by the TMS. They found that, when the TMS pulses were delivered above the resting motor threshold (rMT) and were able to induce hand twitches, the temporal coherence between the TMS pulses and the observed virtual hand twitches induced the embodiment of the virtual hand. In particular, the above rMT procedure was able to replicate the significant difference between synchronous and asynchronous conditions, as measured by subjective questionnaires. No difference between synchronous and asynchronous conditions was found for the proprioceptive drift. On the contrary, when (in a control experiment) the TMS pulses were delivered under the rMT and were not able to induce hand twitches, the temporal coherence between the TMS pulses and the observed virtual hand twitches did not induce the embodiment.
This is a very interesting study, including well-designed main and control experiments and providing clear and novel results in the field. However, I only have a major concern that the authors can take into account for their revision.

1. **My major concern is related to the results interpretation that, in my view, need to be supported by a crucial control experiment. According to the authors, the obtained illusory embodiment is explained by the central stimulation of the hand motor representation in the brain. They compared their results to the Collins et al. 2017's results, showing that invasive stimulation of the primary somatosensory cortex was able to induce the embodiment when synchronous visual stimuli were delivered to the fake hand (and no tactile stimulation was delivered to the real hand). However, a more conservative interpretation can explain their results as a passive movement effect, well known in the motor version of the rubber hand illusion. Indeed, only when involuntary hand twitches were induced by the supra rMT TMS the illusion occurred. Involuntary hand twitches, induced by TMS, can be considered as passive, externally triggered, involuntary hand movements. The subjects are usually aware of the hand twitches, they are able to feel them and to report if they occur or not (see for instance, Bruno et al., The role of**

premotor and parietal cortex during monitoring of involuntary movement: a combined TMS and tDCS study, Cortex 2017). Accordingly, in the present experiment, the TMSq results show that the subjects gave greater ratings for hand movement sensations during SUPRA-T-M1 condition (the one able to induce hand twitches). Thus, even if the authors try to explain away this interpretation suggesting that hand twitches are smaller and shorter than passive movements (I actually do not understand very well the second argument related to the contact between the device and the hand), I think that a control experiment is necessary to support their interpretation. Thus, I strongly suggest to include a control experiment in which hand twitches are induced by a peripheral stimulation. According to the authors' interpretation, we can expect that a peripheral stimulation is not able to induce the virtual hand illusion. They can evaluate different procedures to peripherally induce hand twitches; maybe, they can consider stimulating the median nerve at the wrist. If crucial difference will be found between central (TMS over M1) and peripheral stimulations, they can prove that their results cannot be explained as a passive movement effect but they represent the effect of the central stimulation of the hand motor representation in the brain.

We thank the Reviewer for the comment. We recognize that the part of the discussion related to the interpretation of the role of the hand movements in the illusion may have been misleading. In the previous version of the manuscript, we argued that the movements per se cannot evoke the illusion IF NOT combined with CONGRUENT virtual feedback of a moving virtual hand. This does not exclude a role of peripheral movements. However, our design using synchronous and asynchronous supra-threshold TMS conditions excludes that mere peripheral movements are sufficient to induce the illusion, as they are present in both conditions. We agree with Reviewer 2 that the comparison between supra- and sub- threshold TMS over M1 (experiment 2) deserves a more careful discussion of this point. We did not study (in this project) or claim (in the discussion) that our findings suggest that stimulation of the central hand motor representation alone induces ownership even when peripheral movements are not evoked. Nevertheless, we wanted to introduce another possible explanation related to intensity of stimulation. Besides hand twitches, one could indeed hypothesize that the TMS intensity in the sub-threshold condition was too weak to activate a hand motor representation in M1 to be sufficient to trigger the illusion. At present, we cannot exclude this possibility. Whether a higher intensity of stimulation (e.g. around the resting motor threshold) induces ownership even without inducing any muscle twitches remains an issue for futures studies.

Although, we believe that a comparison between transcranial magnetic and peripheral nerve stimulation as suggested by Reviewer 2 would be interesting in general, it will not be sufficient to disambiguate between the role of the M1 and of the peripheral effects in stimulation induced ownership: peripheral nerve stimulation triggering involuntary hand movements may as well induce illusory ownership if congruent feedback in virtual reality were provided. However, this is beyond the scope of the present work. Finally, adding more control experiments would be in contrast with recommendations of Reviewer 1 asking us to shorten and simplify the manuscript.

Based on these considerations and modifications in the revised version of the discussion (see below), we believe that another experiment is not necessary. In the new version of the manuscript this part of the discussion has been fully re-written in order to clarify our interpretations and underline the role of hand twitches in the illusion (page 25):

In addition, no illusory embodiment was elicited by applying a sub-threshold TMS pulse over M1, that did not evoke any sensory or movement effects at the hand (no MEPs, no muscle twitches), further underlining the selectivity of the obtained embodiment effects for the activation of the hand corticospinal representation. This shows that the illusory ownership in the present study was only triggered if hand twitches are evoked by the TMS and are combined with congruent visual feedback of a virtual hand moving accordingly to the evoked movements (synchronous condition). Alternatively, one could hypothesize that the sub-threshold TMS intensity was not sufficient to activate M1 in order to trigger the illusion (in line with studies showing differences in M1 activation depending on TMS intensity, e.g. Shitara et al. 2011). At present, we do not exclude the possibility that slightly higher sub–threshold stimulation (e.g. around the resting motor threshold) could induce illusory ownership even without inducing muscle twitches, an issue that should be investigated in future studies.

Moreover, following the Reviewer's suggestions, also the part related to the comparison with passive movements has been changed (page 23):

Second, active movements are generally accompanied by voluntary motor commands, by motor planning, and by expectations about the resulting sensory feedback (e.g. Wolpert & Ghahramani, 2000). As these latter aspects were absent (or at least strongly diminished), the present TMS-RHI data show that these features characterizing active physical movements are not necessary to induce hand embodiment. Nevertheless, it is important to acknowledge that the afferent information evoked by TMS-induced movements could be very similar to those evoked during passive movements. These inputs could have a role in triggering the illusion when combined with congruent visual feedback of a moving virtual hand (see below the comparison between supra- and sub- threshold condition).

2nd Editorial Decision 08 January 2018

Dear Dr. Bassolino,

Your resubmitted manuscript has been re-reviewed by the original two reviewers and the process was handled by one of our Section Editors. While reviewer 1 is happy with your revisions, reviewer 2 has lingering concerns that are related to claims and aims of your study. He/she points out that claims should be further toned downed and aims aligned to your design, given the lack of some control conditions. We think these points should be addressable with minor to moderate changes, and invite you to submit a revision.

If you do decide to prepare a re-submission, please also ensure that you supply a 'clean' copy of the manuscript, include a graphical abstract, proof-read the reference list for missing information and replace bar charts with more informative scatter plots or similar(in accordance with EJN guidelines).

If you are able to respond fully to the points raised, we would be pleased to receive a revision of your paper within 30 days.

Thank you for submitting your work to EJN.

Kind regards,

Paul Bolam & John Foxe
co-Editors in Chief, EJN

Reviews:

Reviewer: 1 (Nicholas Holmes, University of Nottingham, UK)

Comments to the Author
The authors have responded very well to my comments and I have no further comments. Many thanks.

Reviewer: 2 (Francesca Garbarini, University of Turin, Italy)

Comments to the Author
I thank the authors for revising their manuscript according to my comments on supra-threshold TMS and passive movements.

In their “response letter”, they acknowledge that “peripheral nerve stimulation triggering involuntary hand movements may as well induce illusory ownership if congruent feedback in virtual reality were provided”. If so, in my view, this can tell us that hand twitches, either induced by central stimulation of M1 (as in their paradigm) or by peripheral stimulation, can induce illusory ownership when combined with congruent visual feedbacks, as in the visuo-motor (passive) version of the RHI. Without this control experiment, this possibility cannot be excluded, and this make weaker the strength of the present results.

The authors also mentioned another possible control experiment: “Whether a higher intensity of stimulation (e.g. around the resting motor threshold) induces ownership even without inducing any muscle twitches remains an issue for futures studies”. I think that this proposed control experiment can perfectly work to control the role of hand twitches that is problematic here.

However, I understand their choice to attenuate their claims instead of performing control experiments. Nevertheless, I think that other parts of the text has to be attenuated and also the aim of the study has to be revised.

With respect to their aims, in their response letter, the authors claimed that. “We did not study (in this project) or claim (in the discussion) that our findings suggest that stimulation of the central hand motor representation alone induces ownership even when peripheral movements are not evoked”. However, in the Introduction and in the Discussion they focused on the central hand motor representation alone. For instance: “One intriguing possibility is to induce embodiment by stimulating not the subject’s body, but directly the brain’s body representation; “… the possibility to induce hand embodiment in healthy participants by stimulating the corticospinal hand representation non-invasively, has been never investigated, so far”; “We hypothesized that the combination between the activation of the hand corticospinal representation (induced through TMS), time-locked with visual feedback provided via VR (synchronous condition), induces illusory embodiment for the virtual limb”; “Inducing illusory embodiment by artificially stimulating the hand corticospinal representation”. The authors can consider to revise these sentences.

In particular, I think that the conclusion of the abstract has to be changed. “This reveals that non-invasive brain stimulation may replace motor-related components (volition, planning, and anticipation of sensorimotor feedback) and the application of external tactile hand cues in embodiment”. Motor-related components also include sensorimotor feedback coming from the induced hand-twitches.

Authors’ Response 15 January 2018

Reviewer: 2

Comments to the Author
I thank the authors for revising their manuscript according to my comments on supra-threshold TMS and passive movements.
In their “response letter”, they acknowledge that “peripheral nerve stimulation triggering involuntary hand movements may as well induce illusory ownership if congruent feedback in virtual reality were provided”. If so, in my view, this can tell us that hand twitches, either induced by central stimulation of M1 (as in their paradigm) or by peripheral stimulation, can induce illusory ownership when combined with congruent visual feedbacks, as in the visuo-motor (passive) version of the RHI. Without this control experiment, this possibility cannot be excluded, and this make weaker the strength of the present results.
The authors also mentioned another possible control experiment: “Whether a higher intensity of stimulation (e.g. around the resting motor threshold) induces ownership even without inducing any muscle twitches remains an issue for futures studies”. I think that this proposed control experiment can perfectly work to control the role of hand twitches that is problematic here.
However, I understand their choice to attenuate their claims instead of performing control experiments. Nevertheless, I think that other parts of the text has to be attenuated and also the aim of the study has to be revised.
1. **With respect to their aims, in their response letter, the authors claimed that. “We did not study (in this project) or claim (in the discussion) that our findings suggest that stimulation of the central hand motor representation alone induces ownership even when peripheral movements are not evoked”. However, in the Introduction and in the Discussion they focused on the central hand motor representation alone. For instance: “One intriguing possibility is to induce embodiment by stimulating not the subject’s body, but directly the brain’s body representation; “… the possibility to induce hand embodiment in healthy participants by stimulating the corticospinal hand representation non-invasively, has been never investigated, so far”; “We hypothesized that the combination between the activation of the hand corticospinal representation (induced through TMS), time-locked with visual feedback provided via VR (synchronous condition),**

induces illusory embodiment for the virtual limb"; "Inducing illusory embodiment by artificially stimulating the hand corticospinal representation". The authors can consider to revise these sentences.

We understand the reviewer's concern. According to the reviewer's suggestion, we have changed the text of the revised manuscript and deleted the statement "by stimulating not the subject's body" that could have been misunderstood and now explicitly mentioned the peripheral movements induced by TMS, as you can read below.

Introduction, page 4:
Thus, most RHI work has been based either on the direct application of somatosensory stimuli to participant's skin or limb (i.e. tactile, Botvinick & Cohen, 1998 or proprioceptive cue, Walsh et al., 2011) or on subjects' movements (Tsakiris et al., 2006; Riemer et al., 2013, 2014; Kalckert & Ehrsson, 2014). One intriguing possibility is to induce embodiment by providing artificial stimulation able to activate the corticospinal hand representation. Despite recent clinical evidence in this direction (in two epileptic patients undergoing invasive stimulation of somatosensory cortex evoking artificial somatic sensations in patients' hand)(Collins et al., 2017), the possibility to induce hand embodiment in healthy participants by artificially activating the corticospinal hand representation non-invasively, that is through transcranial magnetic stimulation (TMS), has been never investigated, so far.
Here, we linked the stimulation of the hand corticospinal representation induced by TMS over the motor cortex (M1) with visual hand feedback provided by an immersive VR system (TMS-VR induced RHI) to investigate embodiment induction for a virtual hand. If applied over hand M1, a TMS pulse, at a sufficient intensity, induces measurable twitches and short involuntary hand movements and in the corresponding hand muscle (i.e. motor evoked potentials, MEPs). In our new experimental set-up, TMS automatically triggered the VR system, so that participants observed an animated virtual hand mimicking the TMS-induced movements. We hypothesized that the combination between the activation of the hand corticospinal representation (induced through TMS), leading to involuntary hand twitches, time-locked with visual feedback provided via VR (synchronous condition), induces illusory embodiment for the virtual limb.

The title "Inducing illusory embodiment by artificially stimulating the hand corticospinal representation" has been changed to (Discussion, page 22): *Inducing illusory embodiment by triggering artificial hand twitches through TMS.*
In addition, some parts of the Discussion have been modified accordingly:

Page 20
The present findings demonstrate that a TMS supra-threshold artificial signal over M1~~induced by TMS in the hand corticospinal representation and~~ triggering hand twitches, when combined with time-locked visual feedback, is able to induce embodiment for a virtual hand in healthy participants.

Page 21
Here, we directly coupled visual hand feedback via a fully controlled VR system with the TMS-induced activation of ~~M1~~ the hand corticospinal tract to induce the RHI.

Page 22
Second, active movements are generally accompanied by voluntary motor commands, by motor planning, and by anticipation ~~expectations about the resulting sensory feedback~~ (e.g. Wolpert & Ghahramani, 2000).

Conclusion
Here, we further extend this concept by showing that is possible to induce embodiment for a virtual hand by activating the corticospinal sensorimotor system via TMS, without motor-related components of volition, planning, or anticipation ~~of sensorimotor feedback~~.
...
We argue that ~~at cortical level~~ illusory embodiment in the present experiments is due to neuro-visual integration between TMS-induced M1 activation (and connections with sensory cortex, e.g. Jacobs et al. 2012) and hand twitches ~~in M1 (and connections with sensory cortex, e.g. Jacobs et al. 2012)~~ with visual VR feedback. ~~and/or neural processing of hand ownership in fronto-parietal networks (Grivaz et al., 2017).~~

EJN European Journal of Neuroscience FENS Federation of European Neuroscience Societies

2. **In particular, I think that the conclusion of the abstract has to be changed. "This reveals that non-invasive brain stimulation may replace motor-related components (volition, planning, and anticipation of sensorimotor feedback) and the application of external tactile hand cues in embodiment". Motor-related components also include sensorimotor feedback coming from the induced hand-twitches.**

This sentence has been changed to: *"This reveals that non-invasive brain stimulation may replace the application of external tactile hand cues and motor components related to volition, planning, and anticipation".*

3rd Editorial Decision 17 January 2018

Dear Dr. Bassolino,

Your revised manuscript was re-evaluated by one of the external reviewers as well as by the Section Editor, Prof. Gregor Thut and ourselves. We are pleased to inform you that it will be accepted for publication in EJN after a couple of minor revisions. In accordance with EJN guidelines, please replace bar charts with more informative scatter plots or similar (see our recent EJN editorial - Rousselet, Foxe and Bolam - http://onlinelibrary.wiley.com/doi/10.1111/ejn.13400/full). Please also submit a 'clean' copy of the manuscript. Once done, we will send it off to the typesetters.

If you are able to respond fully to the points raised, we shall be pleased to receive a revision of your paper within 30 days.

Thank you for submitting your work to EJN.

Kind regards,

Paul Bolam & John Foxe
co-Editors in Chief, EJN

Reviews:

Reviewer: 2 (Francesca Garbarini, University of Turin, Italy)

Comments to the Author
There are no further comments.

Authors' Response 09 February 2018

We would like to thank you, the Editors and the Reviewer for their comments. We have now replaced all the figures with more informative "box plots", and we have updated the captions, accordingly (please see below). We have removed the lower panels of the previous supplementary figure 2 and 3 referring to accelerometric data, because these pictures are no more crucial given that we have reported all data in the text, as suggested in the last (first) revision. A "clean" copy of the manuscript has been submitted.

Sincerely,

Michela Bassolino & Olaf Blanke

Updated captions:
Figure 2. Experiment 1. Supra-threshold TMS over the M1 versus supra-threshold TMS over the vertex
Figure shows results on subjective ratings at the Embodiment-questionnaire (upper panel) in the supra-threshold M1 versus supra-threshold vertex condition (16 subjects).
"Boxes" are based on the first and third quartiles (inter-quartile range, lower and upper "hinges"), the median (line), the largest and the smallest value no further than 1.5 * the inter-quartile range (upper and lower whiskers), data beyond the end of the whiskers (points). While higher ratings to the "embodiment

questions" after synchronous (in the figure, sync) rather than asynchronous (async) condition were reported in supra-threshold M1, no synchronous-asynchronous difference was found in supra-threshold vertex. This suggests that no illusion was induced after vertex stimulation. No synchronous-asynchronous or M1-vertex difference emerged in the control questions. As expected, subjects' ratings on sensations induced by TMS (TMS-questionnaire, lower panel) on the hand (somatosensory sensation on the hand and perception of TMS-induced movements) were different between supra-threshold M1 and supra-threshold vertex conditions, while general TMS sensations related to the somatosensory sensation on the head or the TMS sound clicks were not different between conditions.

Figure 3. Experiment 2. Supra-threshold versus sub-threshold TMS over M1
Figure shows findings related to subjective ratings (Embodiment-questionnaire, upper panel) in the supra-threshold M1 versus sub-threshold M1 condition (16 subjects). "Boxes" are based on the first and third quartiles (inter-quartile range, lower and upper "hinges"), the median (line), the largest and the smallest value no further than 1.5 * the inter-quartile range (upper and lower whiskers), data beyond the end of the whiskers (points).While higher ratings after synchronous (in the figure, sync) rather than asynchronous (async) condition were reported in supra-threshold M1 for the "ownership for the virtual hand" and "agency" component, this was not the case in sub-threshold M1 condition. No synchronous-asynchronous or supra-sub threshold stimulation difference emerged in the control questions. As expected, and similar to experiment 1, subjects' ratings on sensation induced by TMS (TMS-questionnaire, lower panel) on the hand (somatosensory sensation on the hand and perception of TMS induced movements) were different between supra-threshold M1 and sub-threshold M1 conditions, while general TMS sensations related to somatosensory sensation on the head or the TMS sound clicks were not different between conditions.

Supplementary Figure 1. Main experiment. Supra-threshold TMS over motor cortex (supra-threshold M1) time-locked with virtual reality feedback induces embodiment for a virtual hand
Figure shows results on subjective ratings on Embodiment-questionnaire (upper panel) and TMS-questionnaire (lower panel). "Boxes" are based on the first and third quartiles (inter-quartile range, lower and upper "hinges"), the median (line), the largest and the smallest value no further than 1.5 * the inter-quartile range (upper and lower whiskers), data beyond the end of the whiskers (points). Higher ratings (32 subjects) after synchronous (sync, dark grey) than (async, light grey) condition were reported for all embodiment statements, but not for the control questions (upper panel). Subjects' ratings on any sensation induced by TMS were comparable in synchronous and asynchronous conditions, excluding any generic effect of TMS between conditions (lower panel).

Supplementary Figure 2. Motor evoked potentials (MEPs) did not differ in synchronous or asynchronous condition
"Boxes" are based on the first and third quartiles (inter-quartile range, lower and upper "hinges"), the median (line), the largest and the smallest value no further than 1.5 * the inter-quartile range (upper and lower whiskers), data beyond the end of the whiskers (points). The amplitude of MEPs (32 subjects) was comparable in the synchronous (sync, dark grey) and asynchronous (async, light grey) condition when recorded before (PRE) and after (POST) TMS-VR stimulation (supra-threshold M1), as well as during the stimulation (EXP).

Supplementary Figure 3. Motor evoked potentials (MEPs) in experiment 1 and 2
Figure represents the amplitude of motor evoked potentials (MEPs) before (PRE), during (EXP) and after (POST) the TMS-VR stimulation in experiment 1 (supra-threshold M1 versus supra-threshold vertex, upper panel) and in experiment 2 (supra-threshold M1 versus sub-threshold M1, lower panel) (16 subjects). "Boxes" are based on the first and third quartiles (inter-quartile range, lower and upper "hinges"), the median (line), the largest and the smallest value no further than 1.5 * the inter-quartile range (upper and lower whiskers), data beyond the end of the whiskers (points). Differently from supra-threshold M1 stimulation, as expected, MEPs were absent during TMS-VR stimulation in supra-threshold vertex and in sub-threshold M1.
